# Global leaf and root transcriptome in response to cadmium reveals tolerance mechanisms in *Arundo donax* L

**DOI:** 10.1186/s12864-022-08605-6

**Published:** 2022-06-08

**Authors:** Danilo Fabrizio Santoro, Angelo Sicilia, Giorgio Testa, Salvatore Luciano Cosentino, Angela Roberta Lo Piero

**Affiliations:** Department of Agriculture, Food and Environment, University of Catania, Via Santa Sofia 98, 95123 Catania, Italy

**Keywords:** *Arundo donax* L., Bioenergy crops, RNA-seq, De novo assembly, Leaf and root transcriptome, Heavy metals, Cadmium

## Abstract

The expected increase of sustainable energy demand has shifted the attention towards bioenergy crops. Due to their know tolerance against abiotic stress and relatively low nutritional requirements, they have been proposed as election crops to be cultivated in marginal lands without disturbing the part of lands employed for agricultural purposes. *Arundo donax* L. is a promising bioenergy crop whose behaviour under water and salt stress has been recently studied at transcriptomic levels. As the anthropogenic activities produced in the last years a worrying increase of cadmium contamination worldwide, the aim of our work was to decipher the global transcriptomic response of *A. donax* leaf and root in the perspective of its cultivation in contaminated soil. In our study, RNA-seq libraries yielded a total of 416 million clean reads and 10.4 Gb per sample. De novo assembly of clean reads resulted in 378,521 transcripts and 126,668 unigenes with N50 length of 1812 bp and 1555 bp, respectively. Differential gene expression analysis revealed 5,303 deregulated transcripts (3,206 up- and 2,097 down regulated) specifically observed in the Cd-treated roots compared to Cd-treated leaves. Among them, we identified genes related to “Protein biosynthesis”, “Phytohormone action”, “Nutrient uptake”, “Cell wall organisation”, “Polyamine metabolism”, “Reactive oxygen species metabolism” and “Ion membrane transport”. Globally, our results indicate that ethylene biosynthesis and the downstream signal cascade are strongly induced by cadmium stress. In accordance to ethylene role in the interaction with the ROS generation and scavenging machinery, the transcription of several genes (NADPH oxidase 1, superoxide dismutase, ascorbate peroxidase, different glutathione S-transferases and catalase) devoted to cope the oxidative stress is strongly activated. Several small signal peptides belonging to *ROTUNDIFOLIA*, *CLAVATA3*, and C-TERMINALLY ENCODED PEPTIDE 1 (CEP) are also among the up-regulated genes in Cd-treated roots functioning as messenger molecules from root to shoot in order to communicate the stressful status to the upper part of the plants. Finally, the main finding of our work is that genes involved in cell wall remodelling and lignification are decisively up-regulated in giant reed roots. This probably represents a mechanism to avoid cadmium uptake which strongly supports the possibility to cultivate giant cane in contaminated soils in the perspective to reserve agricultural soil for food and feed crops.

**Supplementary Information:**

The online version contains supplementary material available at 10.1186/s12864-022-08605-6.

## Introduction

Nowadays, globe climate change has become one of the most urgent problems humans have to deal with due to the ongoing Green House Gas (GHG) emissions into the atmosphere. Fossil fuels based on organic origin and non-renewable energy sources are by now unsustainable, thereby new energy policies should be considered in order to meet the global energy demand [1]. Among the different renewable energy sources, second generation of biofuels (biomass) is becoming an interesting candidate because of its peculiarity to be converted in a variety of products, such as solid fuels, liquid fuels, heat, electricity, and hydrogen. In particular, biomass is mainly produced from organic origin by using perennial herbaceous plants and fast-growing trees, which are able to limit GHG levels [2, 3]. Bioenergy crops have the capability to be cultivated on marginal land, this fact being very attractive in order not to compete with feed and food crops for land use [4]. This fact assumes a crucial importance in the perspective that the global world’s population is expected to reach 9.7 billion by 2050 [5], thus determining a considerable increase for agricultural land demand [6]. Such marginal sites have little or no agricultural or industrial value, are characterized by little potential for profit, and often have poor soil or other undesirable characteristics such as high salt content, inadequate water supply or heavy metal (HM) contaminations. Although some HMs are categorized as essential elements because of their positive impact on the plant growth and crop yield (e.g., B, Cu, Co, Fe, Mn, Ni, Zn), some of them, such as Cd, Hg, Pb and As, do not play any role in plant metabolism and can reduce the crop productivity in case their levels reach toxic concentrations [7]. Given both its high toxicity and the high capacity of diffusion in lands, cadmium has led to contaminated soils worldwide [8, 9] and in particular in Europe where its concentration in the topsoil raised from < 0.01 to 14.1 ppm in the last years [10]. Moreover, due to its high solubility in soil, cadmium can be easily extracted by the plant root system [11], from where it is accumulated in the above-ground regions inhibiting plant growth and causing a threat to animal and human health through the food chain [12]. Cadmium accumulation-related molecules include many transporters, chelators, some amino acids and organic acids, and the genes encoding the corresponding proteins/enzymes were functionally characterized in both *A. thaliana* and *O. sativa* [13]. A group of transmembrane proteins involved in metal transport and homeostasis is represented by the family of NRAMP metal ion transporters which is supposed to be the major transporter family of Cd^2+^ from the soil into root cells [14]. Once inside the cells, the mechanisms by which Cd exerts toxicity include: a) substitution of some essential metal ion (e.g. Zn^2+^ and Fe^2+^) or blocking functional groups which leads to inactivation of biomolecules [15], b) a tight binding with thiol groups of proteins which destroys their structure and function [16], and c) generation of reactive oxygen species (ROS), which brings to oxidative stress [17, 18].

*Arundo donax L.* also known as giant reed, is a perennial rhizomatous grass species, genus *Arundo*, belonging to *Poaceae* family. Among rhizomatous grasses dedicated to energy production, *A. donax* represents one of the most promising bioenergy crops [19, 20] because of its high biomass production, both low irrigation and nitrogen input requirements, and its high tolerance to abiotic stress conditions, including herbicide, salinity and heavy metals [21, 22]. Notably, it has been proposed as species to be employed for phytoremediation [23] due to its ability to accumulate and tolerate high doses of heavy metals, such as Ni, Cd and As [24, 25].

Transcriptomic analysis represents a powerful tool to elucidate the molecular mechanisms by which plants accumulate, translocate and detoxify HMs [26]. Moreover, RNA Sequencing (RNA-Seq) and de novo assembly of the transcriptome allow the discover and the quantitative determination of all the expressed genes for those species whose genome sequence is not available yet, such as *A. donax*. Existing genomic resources of *A. donax* were provided by RNA sequencing extracted from different organs (leaf, culm, bud and root) of giant reed ecotypes subjected to either normal [27–29] or under water stress condition [30]. More recently, the analysis of the transcriptional response of giant reed was performed after a long-term period of salt stress in two different *A. donax* ecotypes [31, 32]. Overall, these studies contributed both to the drafting of candidate gene list that can be used in molecular breeding projects and in revealing the role of plant genes in the abiotic stress response. However, the transcriptional response of *A. donax* subjected to cadmium treatments is still incomplete and the molecular mechanism of cadmium stress on giant reed metabolic processes remains poorly understood. Recently, Shaheen et al. [33] evaluated the effects of increasing concentrations of cadmium on the expression of selected genes (carotenoid hydroxylase, amidase, glutathione reductase, bHLH, NRAMP and YSL) in *Arundo donax* L. cultivated in hydroponic solution. The highest expression for these genes was observed in plants exposed to the highest Cd concentration (100 mg/L). Moreover, the activity of several enzymes involved in the ROS scavenging (SOD, CAT, POD) was also measured revealing their activation at the highest cadmium concentration and confirming the onset of a secondary oxidative stress [33]. A study conducted in cohorts of *A. donax* strongly suggests that phytochelatin encoding genes (*Ad*PCS1, *Ad*PCS2, and *Ad*PCS3) most likely contribute to Cd detoxification in *A. donax* and the presence of multiple PCS isoforms seems to be advantageous both to provide higher general levels of phytochelatin biosynthesis and to increase flexibility in HM resistance [34].

In this study, considering the frequency of cadmium in soils of Mediterranean basin and the perspective of potential use of marginal land to cultivate bioenergy crops, we sequenced and de novo assembled the giant reed leaf and root transcriptomes after a prolonged period of cadmium treatment by using RNA-Seq technique. The aim of our work was to gain novel insight into the cadmium stress tolerance and to shed new light on the distinct role of leaf and root in the dynamics of heavy metal stress response in plants.

## Material and methods

### Plant material and application of cadmium nitrate

The experiment was conducted at the Department of Agricultural, Food and Environment (Di3A) of the University of Catania, using G10 ecotype coming from Fondachello (Italy) (N 37^o^45, E 15^o^11’), collected for the Giant reed Network project [35]. The trial started on May 4^th^ 2020, by filling each pot with 8 kg of clay soil and kept in open air. The contamination of each pots was achieved by using 4 ppm (4 mg/kg) of cadmium nitrate Cd(NO_3_)_2_ for treated plants and 5.774 g of NH_4_NO_3_ for control plants, so as to equilibrate the N concentration between the two conditions. The aforementioned concentration is higher than the reference threshold established by the European Union directive 86/278/EEC on Environment protection which establishes the maximum concentration of cadmium on soil should range between 1 and 3 ppm [36]. Cadmium contents above 3 mg/kg are generally thought to indicate contaminated soil [37]. Afterwards, one litre of tap water was added to each pot to allow the element adsorption by soil colloids. Successively, in each pot a single *A. donax* stem node was transplanted and the irrigation was performed three times a week by adding one litre of tap water to avoid Cd(NO_3_)_2_ leaching. The pots were arranged according to a randomized block factor scheme, considering three biological replicates for each treatment, with a total of six experimental units. Before sampling (July 28^th^, 2020, after three months from the start of the trial), the following morpho-biometric and physiological parameters were evaluated: number of culms per pots, height of the main culm, net photosynthesis efficiency measured by LCi-T portable photosynthesis system (ADC BioScientific Ltd). Moreover, the measurement of biomass was performed [35]. R software was used for standard deviation analysis. The data were submitted to one-way ANOVA test using R Studio. The averages with *p*value ≤ 0.05 were separated by the TukeyHSD test.

### Sample collection and RNA extraction

On July 28^th^ 2020, fully expanded, no senescing G10 leaves and roots were harvested and immediately frozen with liquid nitrogen [32]. RNA isolation was carried out by using the Spectrum Plant Total RNA Extraction kit (Sigma-Aldrich, St. Louis, MO, USA) according to the manufacturer’s instructions. RNA degradation and contamination were monitored by electrophoresis with 1% agarose gel. RNA purity and concentration were assayed using the NanoDrop spectrophotometer (ThermoFisher Scientific, Waltham, MA, USA) [32]. Before to be sequenced, the RNA samples were subjected to quality parameter evaluation. RNA integrity was assessed using the Agilent Bioanalyzer 2100 system (Agilent Technologies, Santa Clara, CA, USA) [31].

### Library preparation for transcriptome sequencing

One µg of RNA was used as input material for library preparations (twelve libraries, one for each sample). Sequencing libraries were generated using NEBNext ® Ultra ™ RNA Library Prep Kit for Illumina® (New England Biolabs, Ipswich, MA, USA) following manufacturer’s recommendations [31]. Briefly, mRNA was purified from total RNA using poly-T oligo-attached magnetic beads. Fragmentation was carried out using divalent cations under elevated temperature in NEBNext First Strand Synthesis Reaction Buffer (5X). First strand cDNA was synthesized using random hexamer primer and M-MuLV Reverse Transcriptase (RNase H) as synthesizing enzyme [31]. Second strand cDNA synthesis was subsequently performed using RNase H to insert breaks into the RNA molecule and DNA Polymerase I as synthesizing enzyme. Remaining overhangs were converted into blunt ends via exonuclease/polymerase activities. After adenylation of 3′ ends of DNA fragments, NEBNext Adaptor with hairpin loop structure were ligated to prepare for hybridization. In order to select cDNA fragments of preferentially 150 ~ 200 bp in length, the library fragments were purified with AMPure XP system (Beckman Coulter, Beverly, MA, USA). Then 3 μl USER Enzyme by NEB were used with size-selected, adaptor-ligated cDNA at 37 °C for 15 min followed by 5 min at 95 °C before PCR. Then PCR was performed with Phusion High-Fidelity DNA polymerase, Universal PCR primers and Index (X) Primer. Finally, PCR products were purified (AMPure XP system) and library quality was assessed on the Agilent Bioanalyzer 2100 system [31].

### Clustering and next generation RNA sequencing

Cluster generation and sequencing were performed by Novogene (UK) company Limited (25 Cambridge Park, Milton Road, Cambridge, CB4 OFW, United Kingdom). The clustering of the index-coded samples was performed on a cBot Cluster Generation System using a PE Cluster kit cBot-HS (Illumina) [31]. After cluster generation, the library preparations (twelve libraries, one for each sample) were sequenced on Illumina HiSeq2000 platform to generate paired-end reads whose size was paired-end 2 × 150 bp reads. Raw reads in fastq format were firstly processed through in-house perl scripts. In this step, clean data were obtained by removing reads containing adapters, reads containing poly-N and low-quality reads [31]. At the same time, Q20, Q30, GC-content and sequence duplication level of the clean data were calculated. All the downstream analyses were based on clean data with high quality (Table 1) [31].

**Table 1:** Summary statistics of the RNA quality and sequencing results

Average RIN	7.05
Raw data	430 million
Clean reads	416 million
Adapter related	14 million
N^o^ Transcripts	378,521
N^o^ Unigenes	126,668
Mapping rate	69.0
Transcript N50 (bp)	1,812
Unigenes N50 (bp)	1,555
Q30 (%)	94.37
GC content (%)	55.08

### De novo assembly and gene functional annotation

De novo transcriptome assembly was made up by Trinity software (2.6.6 version) with min_Kmer_Cov = 3 and min_glue = 4 [38]. Hierarchical Clustering was carried out by Corset (4.6 version) in order to remove redundancy (parameter -m 10), so that the longest transcript of each cluster has been selected as Unigene [39]. The assembly assessment and gene prediction have been performed by Benchmarking Universal Single-Copy Orthologous (BUSCO software, 3.0.2 version) [40], whereas the gene functional annotation was obtained by exploiting seven different databases: National Centre for Biotechnology Information (NCBI), non-redundant protein sequences (Nr, Diamond software, 0.8.22 version, e-value threshold 1e-5) [41], NCBI non-redundant nucleotide sequences (Nt, NCBI blast software, 2.9.0 version, e-value threshold 1e-5), Protein family (Pfam, hmmscan software, HMMER 3.1 version, e-value threshold 0.01) [42], Cluster of Orthologous Groups of Proteins (KOG/COG, Diamond software, 0.8.22 version, e-value threshold 1e-5) [41], Swiss-Prot (Diamond software, 0.8.22 version, e-value threshold 1e-5) [41], Kyoto Encyclopedia of Genes and Genome (KEGG, Diamond and KAAS software, 0.8.22 version, e-value threshold 1e-5) [41, 43] and Gene Ontology (GO, blast2GO software, b2g4pipe_v2.5 version, e-value threshold 1e-6) [44]. To identify the transcription factor, iTAK (hmmerscan software) [42] tool was used to infer the TF families [45, 46].

### Quantification of gene expression and differential expression analysis

Gene expression level was estimated by RSEM software (1.2.28 version) by mapping back each clean read onto assembled transcriptome and read counts for each gene were then obtained from the mapping results [47]. Furthermore, the read counts of each gene have been used as input data for DESeq2 (1.26 version, padj ≤ 0.05), to obtain differentially expressed genes (DEGs) [48]. Comparisons were made to identify the set of differentially expressed genes between control (CK) and cadmium (Cd) treatments for each tissue (Cd_L_vs CK_L; Cd_R vs CK_R). Moreover, the core of our analysis was performed on the comparison between the Cd-treated samples of the two tissues indicated as Cd_R vs Cd L*. In this comparison, the Cd-treated root vs Cd-treated leaf DEGs (Cd_R vs Cd L*) were obtained by subtracting the DEGs belonging to the CK_R vs CK_L comparison (tissue specific DEGs in control conditions) to the Cd_R vs Cd_L. An adjusted *p*-value cutoff of 0.05 and a log_2_fold change (Log_2_FC) threshold of 1 was adopted to filter the significantly up- and down-regulated genes. A correlation analysis was performed in order to demonstrate experiment repeatability and to reveal differences in gene expression among samples. Principal Component Analysis 3D plot and a heatmap were obtained by using R language, considering the read counts of each sample, including biological replicates, as input data.

### Real-time validation of selected DEG candidates using qRT-PCR

Total RNA (2.5 µg) purified from leaves and roots as described above, was reverse transcribed using SuperScript™ Vilo™ cDNA synthesis kit by ThermoFischer Scientific, according to manufacturer’s instructions. Real-time qRT-PCR was carried out for a total of 8 DEGs with PowerUp SYBR Green Master mix by ThermoFischer Scientific in the Bio-Rad iQ5 Thermal Cycler detection system. All the genes have been normalized with *cyclin-dependent kinase C-2* (CDKC-2, XM_004962139), being a suitable housekeeping gene [49]. All reactions were performed in duplicate and fold change measurements calculated with the 2^−∆∆CT^ method.

### Gene ontology and KEGG enrichment analysis

Based on differentially expressed genes (DEGs), the GO enrichment was accomplished by using blast2go (b2g4pipe_v2.5 version) software (e-value = 1e-6) [44]. Furthermore, to analyse the *Arundo donax* L. transcriptome, all the unigenes were submitted to KEGG database for the systematic analysis of gene function. KOBAS software (v.3.0, corrected *p*-value ≤ 0.05) has been applied to test the statistical enrichment of differentially expressed genes in KEGG pathway [50]. Moreover, a pathway analysis was conducted using MapMan3.6.0RC1 (https://mapman.gabipd.org/) [51]. All the unigenes were annotated and mapped using Mercator4 V2.0, an on-line tool of MapMan (https://www.plabipd.de/portal/mercator4) which accurately assigns hierarchal ontology providing visual representation of genes in different plant processes. The significant DEGs (padj < 0.05), with the corresponding log2FoldChange values, were used as dataset to align with the Mercator map. In order to analyse the main gene families and pathways affected by cadmium treatment, DEGs (Cd_R vs Cd_L* comparison) belonging to “Phytohormone action”, “Transcription factors”, “Nutrient uptake”, “Cell wall”, “Polyamine metabolism”, “ROS scavenging” and “Heavy metal transporter” categories were aligned to the *Arabidopsis thaliana* genome (Phytozome genome ID: 167, NCBI taxonomy ID: 3702) accessed through Phytozome 13 (https://phytozome-next.jgi.doe.gov/info/Athaliana_TAIR10). The DEGs showing a threshold of + / − 2.000 Log_2_FoldChange and an e-value ≤ 0.05 were selected.

### KEGG classification of cadmium and salt stress common genes

In order to elucidate the core response of *A. donax* to both salt [31, 32] and cadmium stress, the significant DEGs (padj ≤ 0.05) belonging to both S3_vs_CK (specifically deregulated DEGs under severe salt stress, 256.67 mM NaCl), and S4_vs_CK (specifically deregulated DEGs under extreme salt stress, 419.23 mM NaCl) comparisons [31, 32] were filtered considering a threshold of ± 2.00 log2FoldChange and merged, thus resulting in a “list of salt DEGs”. The same procedure was adopted to retrieve the significant DEGs (padj ≤ 0.05) belonging to Cd_R vs Cd_L*, Cd_L vs Cd_CK and Cd_R vs Cd_CK comparisons, resulting in a “list of cadmium DEGs”. A KO ID (KEGG ORTHOLOGY Database, https://www.genome.jp/kegg/ko.html) was assigned to each DEG, then, the two lists were compared by using the KO ID as common annotation code. The aforementioned comparison generated a merged DEG list where only DEGs whose KO ID was in both the “list of salt DEGs” and “list of cadmium DEGs” were included. Finally, genes were grouped by concordant type of regulation (up-regulated or down-regulated in both stresses).

## Results

### Effect of cadmium upon *A. donax* morpho-biometric and physiological parameters

As described in the Material and Methods section, both morpho-biometric and physiological parameters of *A. donax* G10 ecotype were evaluated at sampling date after being subjected to 4 ppm (4 mg/kg) of cadmium nitrate. A picture of giant reed G10 ecotype at sampling time is shown in Supplementary file 1: Figure S1. Considering the average values of the three biological replicates, we observed that both the main stem height and biomass dry weight per pot were reduced in those samples subjected to Cd treatment (Supplementary file 2: Figure S2A and S2B). Moreover, the net photosynthesis efficiency was also significantly reduced in treated samples compared to the untreated samples, reaching values of 8.64 µmol CO_2_ m^−2^ s^−1^ and 14.91 µmol CO_2_ m^−2^ s^−1^, respectively (Supplementary file 2: Figure S2C). The alteration of the aforementioned parameters indicated the effectiveness of the cadmium dose to induce response in G10 giant reed ecotype.

### Transcript assembly and annotation

In this study, the RNASeq approach was used to comprehensively identify the transcriptional response of the *A. donax* G10 ecotype in leaves and roots. A flow chart of the giant reed leaf and root transcriptome sequencing and de novo assembly process is shown in Supplementary File 3: Figure S3. RNA integrity was checked prior sequencing and the average RNA integrity number (RIN) was 7.05. This shows that all samples were of good quality for further processing and sequencing (Table 1). After sequencing, we filtered the raw reads to remove the adapter-based or poor quality reads, obtaining a total of clean reads equal to 416 million (Table 1) representing the 96.89% of the total reads. Downstream analysis was then performed on about 34.7 million reads (10.4 Gb) per sample, for a total of 416 million clean reads, with Q30 and GC at 94.37% and 55.08%, respectively (Table 1). The clean read de novo assembly yielded 378,521 transcripts and 126,668 unigenes with N50 lengths of 1812 bp and 1555 bp, respectively (Table 1), consistent with previously reported N50 values ​​[29–32]. To assess assembly consistency, filtered unique reads were mapped to the reconstructed transcriptome and average read mapping rate using bowtie2 alignment software was equal to 69.0% (Table 1). The completeness of the assembled transcriptome was evaluated by comparing it to the set of *Embryophyta* genes using the BUSCO quality assessment tool coupled with the OrthoDB (9.0 version) database of orthologs [40]. Among the searched 1440 BUSCO groups, 68.54% (987 BUSCOs) were complete (912 single-copy orthologs and 75 duplicated), 20.0% (288 BUSCOs) were fragment and 11.5% (165 BUSCOs) were missing. These results are comparable to those obtained previously in *A. donax* (70.07%, 13,19% and 16.74%, respectively) [29]. In addition, both transcript and unigene length distributions were reported (Supplementary file 4: Figure S4). Consequently, these results indicated that the sequencing quality was reliable to perform downstream analysis.

Functional annotation of the *A. donax* Unigenes was conducted by performing BLAST searches against public databases, such as the National Center for Biotechnology Information (NCBI), Protein Family (Pfam), Protein Ortholog Group Clusters (KOG / COG), SwissProt, Ortholog Database (KO), Gene Ontology (GO) (Table 2). A total of 88,139 unigenes were annotated in at least one database, and the frequency of Unigenes annotated in at least one searched database was 69.58%. Among them, 73,588 (58.08%) and 63,917 (50.46%) unigenes showed identity with the sequences in the Nr and Nt databases, respectively. The distributions of unigenes homologous to the sequences in the KO, SwissProt, Pfam, GO, and KEGG databases were 21.67, 40.83, 41.54, 41.54, and 13.71%, respectively (Table 2).

**Table 2:** The number and frequency of successful annotated genes

Database	Number of unigenes	Frequency %
Annotated in NR	73,588	58.09
Annotated in NT	63,917	50.46
Annotated in KO	27,461	21.67
Annotated in SwissProt	51,724	40.83
Annotated in PFAM	52,630	41.54
Annotated in GO	52,627	41.54
Annotated in KOG	17,370	13.71
Annotated in at least one database	88,139	69.58

### Identification of differentially expressed genes (DEGs)

**Fig. 1:**
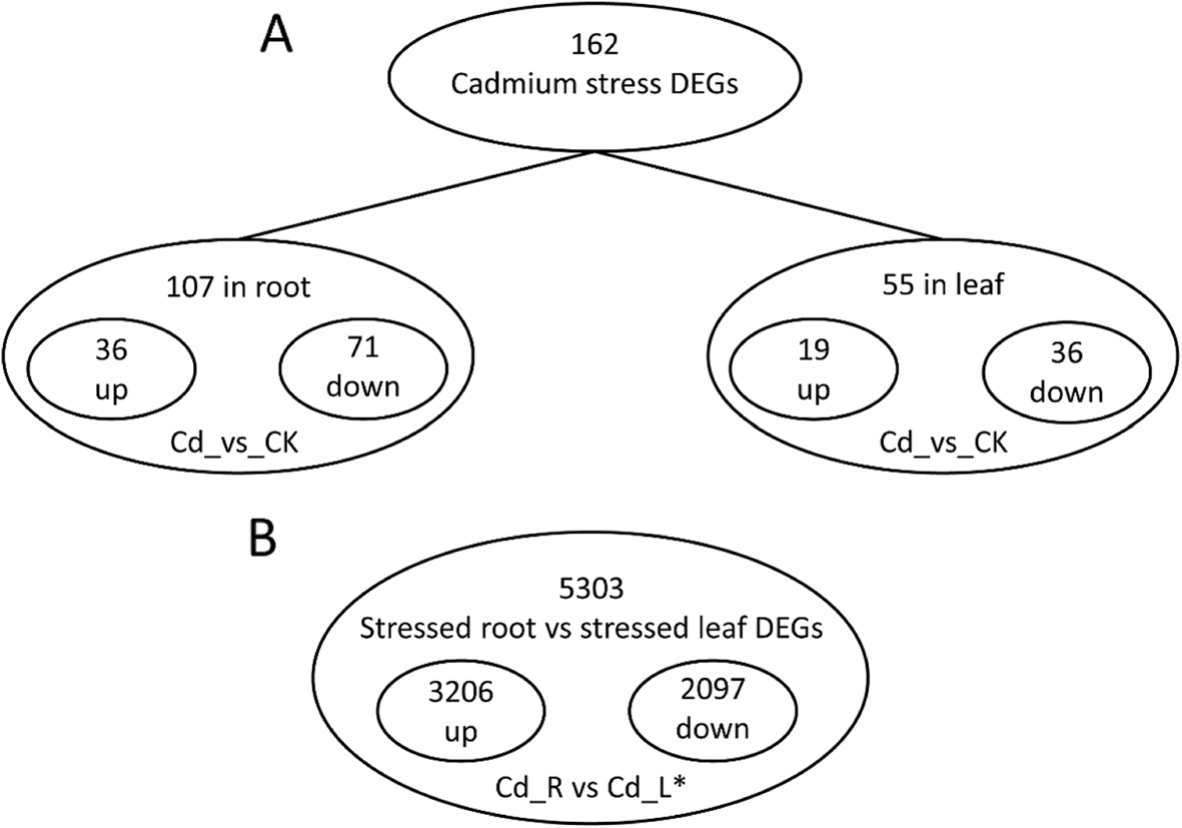
Summary of DEGs in roots and leaves of *A.donax* upon cadmium treatment. **A** Number of up-/down-regulated genes by Cd in different tissues (root treated vs control samples; leaf treated vs control samples). **B** Number of regulated genes between different tissues in stressed conditions. * The stressed root vs stressed leaf DEGs were obtained by subtracting the DEGs belonging to the CK_R vs CK_L comparison to the Cd_R vs Cd_L comparison

The characterization of root and leaf *A. donax* transcriptome was accomplished by the identification of those unigenes whose expression level changed upon cadmium treatment. Based on the experimental design, a total of 162 genes showed differential expression in response to Cd treatment, 107 of them differentially expressed in root (Cd_R vs CK_R) and the remaining 55 differentially expressed in leaf (Cd_L vs CK_L). Among the 107 DEGs found in root, 36 were up-regulated and 71 down-regulated. In leaf, 19 genes resulted up-regulated and 36 down-regulated (Fig. 1A). No DEGs were found in common between root and leaf. The higher number of DEGs in root with respect of the leaf suggested that root, representing the interface between soil and plant, is subjected to a wider reprogramming of the gene expression than leaves (Supplementary file 5; Table S1). DEGs identified in biological replicates clustered together in both organs, indicating good reproducibility of treatment (Supplementary file 6; Figure S5). Moreover, samples either belonging to control or treated samples clustered very close, thus probably explaining the low number of DEGs retrieved when comparing stressed and control samples (Supplementary file 6; Figure S5, S6). On the contrary, a total of 5303 genes were counted as differentially expressed when the Cd_R vs Cd_L* comparison was analyzed (Fig. 1B). This result was obtained by subtracting the DEGs found in the comparison CK_R vs CK_L (all genes that are differentially expressed because they are tissue specific and not related to cadmium treatment) to the total DEGs retrieved by the comparison Cd_R vs Cd_L. Among the 5303 DEGs, 3206 were up-regulated (showing a higher expression in root than in leaf) and 2097 were down-regulated (showing a lower expression in root than in leaf). Validation of RNAseq experiment was performed by measuring the expression levels of eight selected DEGs by quantitative real-time PCR (qRT-PCR) (Supplementary file 7: Figure S7). The results show high congruence between RNA-Seq and qRT-PCR (coefficient of determination R^2^ = 0.91), which accounts for the high reliability of RNA-Seq quantification of gene expression.

**Fig. 2:**
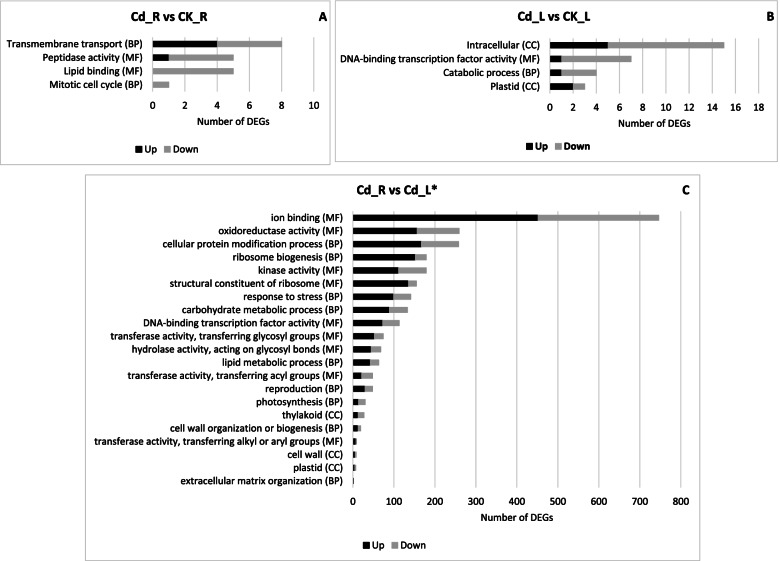
Gene Onthology (GO) enrichment for the DEGs of (**A**) Cd_R vs CK_R, (**B**) Cd_L vs CK_L and (**C**) Cd_R vs Cd_L*. The X-axis indicates the number of DEGs and the Y_axis indicates the subcategories. BP: Biological Process; MF: Molecular Function; CC: Cellular Component

### Functional classification of DEGs

**Fig. 3:**
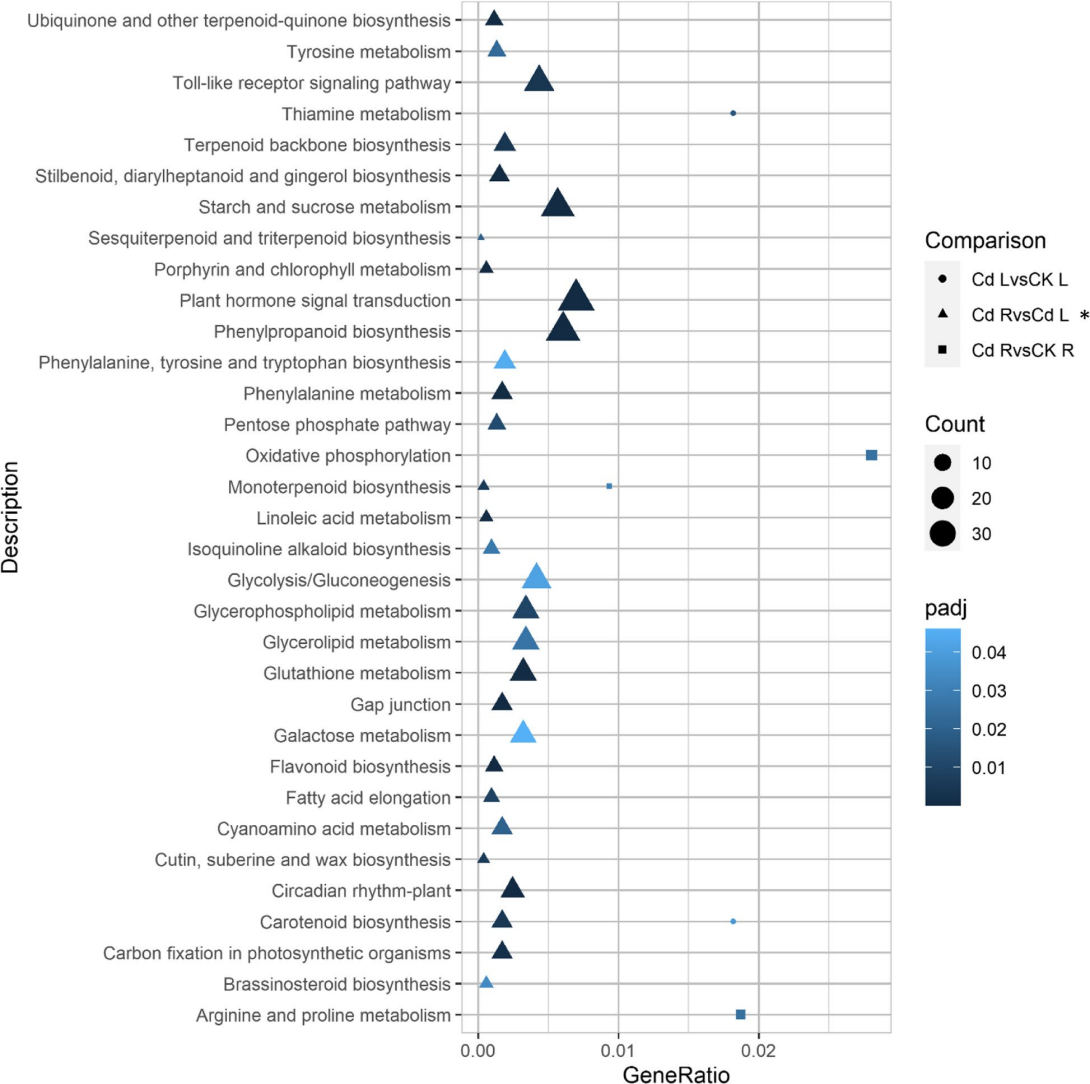
Distribution of Kyoto Encyclopedia of Genes and Genomes (KEGG) pathways for differential expressed genes (DEGs) in the Cd_R vs CK_R, Cd_L vs CK_L and Cd_R vs Cd_L* comparisons (www.kegg.jp/kegg/kegg1.html)

Gene Ontology (GO) terms, Clusters of Orthologous Groups of protein (KOG) classification and Kyoto Encyclopedia of Genes and Genomes (KEGG) pathway functional enrichment were carried out to identify biological processes or pathways involved in cadmium stress response. Considering Cd_R vs CK_R dataset (Fig. 2A), “transmembrane transport” (GO:0,055,085) (4 up- and 4 down-regulated genes), “peptidase activity” (GO:0,008,233) (1 up- and 4 down-regulated genes and “lipid binding” (GO:0,008,289) (0 up- and 5 down-regulated genes) are the three most enriched GO terms. “Intracellular” (GO:0,005,622) (5 up- and 10 down-regulated genes), “DNA-binding transcription factor activity” (GO:0,003,700) (1 up- and 6 down-regulated genes) and “catabolic process” (GO:0,009,056) (1 up- and 3 down-regulated genes) are the most enriched GO terms of Cd_L vs CK_L comparison (Fig. 2B). As concerns the Cd_R vs Cd_L* sample data set (Fig. 2C), “ion binding” (GO:0,043,167) (451 up- and 295 down-regulated genes), “oxidoreductase activity” (GO:0,016,491) (156 up- and 103 down-regulated genes), “cellular protein modification process” (GO:0,006,464) (167 up- and 91 down-regulated genes), “ribosome biogenesis” (GO:0,042,254) (152 up- and 27 down-regulated genes) and “kinase activity” (GO:0,016,301) (111 up- and 68 down-regulated genes) are the most enriched GO terms. To predict any possible functions, all identified unigenes (126,668) were aligned to the KOG database to assign their corresponding KOG category (Supplementary File 8: Figure S8). Among the KOG categories, those clusters encoding for “Translation, ribosomal structure and biogenesis” (15.24%), “Post-translation modification, protein turnover, chaperones” (14.38%), “General function predict only” (11.59%) represented the largest group, followed by “Intracellular trafficking, secretion, and versicular transport” (6.88%), “Energy production and conversion” (6.84%), “Signal transduction mechanisms” (6.67%), “RNA processing and modification” (5.49%), “Amino acid transport and metabolism (5.25%), “Carbohydrate transport and metabolism” (4.68%), “Transcription” (4.55%) and “Lipid transport and metabolism (4.42%) were the most numerous categories (Supplementary File 8: Figure S8). The sets of DEGs originated from the above-described three comparisons (Cd_R vs CK_R, Cd_L vs CK_L and Cd_R vs Cd_L*) were mapped onto KEGG enrichment pathways. The main KEGG pathway terms were plotted in the Fig. 3. Considering both Cd_R vs CK_R and Cd_L vs CK_L sample data sets, we observed pathways represented by few DEGs each: “Oxidative phosphorylation”, “Arginine and proline metabolism” and “Monoterpenoid biosynthesis” in Cd_R vs CK_R comparison, and “Thiamine metabolism” and “Carotenoid biosynthesis” in Cd_L vs CK_L comparison (Fig. 3). As concerning Cd_R vs Cd_L* comparison “Plant hormone signal transduction”, “Phenylpropanoid biosynthesis”, “Starch and sucrose metabolism”, “Toll-like receptor signaling pathway” and “Glycolysis/Gluconeogenesis” were the five most represented KEGG pathways (Fig. 3).

### Identification of transcription factor families involved in plant response to cadmium

**Fig. 4:**
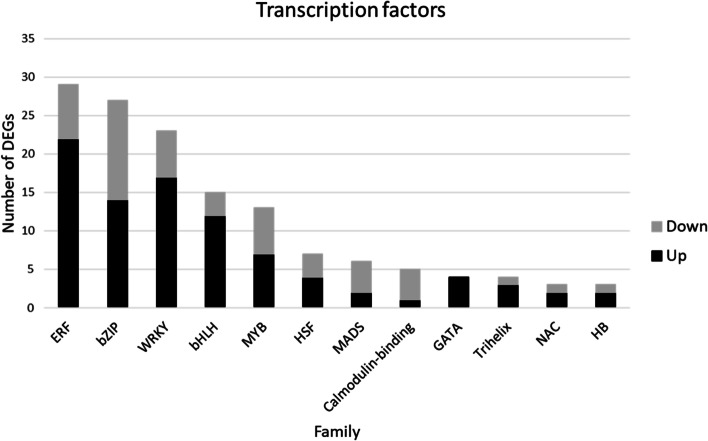
DEGs encoding for TFs. The bars represent the overall TFs found in the three comparisons (Cd_R vs CK_R, Cd_L vs CK_L and Cd_R vs Cd_L*)

Transcription factors (TFs) have been identified as candidate targets for ameliorating plant tolerance in case of cadmium treatment. Therefore, DEGs encoding for TFs were retrieved from each comparison (Cd_R vs CK_R, Cd_L vs CK_L and Cd_R vs Cd_L*), grouped according to the belonging family and sorted for their corresponding abundance (Fig. 4). As result of the analysis, the five more abundant transcription factor families resulted “Ethylene responsive transcription factor” (ERF) (29 DEGs), “bzip” (27 DEGs), “WRKY” (23 DEGs), “bHLH” (15 DEGs) and “MYB” (13 DEGs) (Fig. 4). When different comparisons were considered, two bHLH (both up-regulated) and two ERF (1 up- and 1 down-regulated) TFs were found in the Cd_R vs CK_R dataset and one ERF, two bZIP and one WRKY (all down-regulated) were found in the Cd_L vs CK_L dataset. On the contrary, a higher number of DEGs were found in the Cd_R vs Cd_L* dataset: 26 DEGs belonging to ERF family (21 up- and 5 down-regulated), 25 to bZIP family (14 up- and 11-down regulated), 22 to WRKY family (17 up- and 5 down-regulated), 13 to bHLH family (10 up- and 3 down-regulated) and 13 to MYB family (7 up- and 6 down-regulated).

### Main Processes affected by cadmium treatment

To have a comprehensive view of the metabolic changes occurring in *A. donax* L. under Cd treatment, all the significant DEGs of the Cd_R vs Cd_L* comparison were mapped to the MapMan 3.5.1R2 pathways. The analysis indicated that the five most enriched metabolic pathways are “Protein biosynthesis”, “Phytohormone action”, “Nutrient uptake”, “Cell wall organisation” and “Polyamine metabolism” (Table 3).

**Table 3:** Number of DEGs of the Cd_R vs Cd_L* comparison assigned to the top five MapMan 3.5.1R2 pathways. The significant DEG assignment is indicated by *p*value ≤ 0.05

Pathway Mapman	n° of DEGs	Up	Down	*p*value
Protein biosynthesis	130	128	2	3.25 × 10^–23^
Phytohormone action	56	45	11	7.61 × 10^–06^
Nutrient uptake	18	15	3	1.23 × 10^–03^
Cell wall organisation	17	15	2	1.17 × 10^–02^
Polyamine metabolism	4	0	4	4.80 × 10^–02^

The DEGs belonging to these pathways resulted mostly up-regulated in treated root compared to the treated leaf (Cd_R vs Cd_L*), except for genes of “Polyamine metabolism” that resulted all down-regulated in root compared with the leaf. In addition, considering their crucial role in heavy metal stress response, the coding sequence of each transcripts belonging to “Oxidative stress”, “Heavy metal ATPase 3 (HMA3) transporters” and “NRAMP transporters” categories along with the clusters resulted from the MapMan analysis (Table 3) have been aligned against the *Arabidopsis thaliana* genome (TAIR10) and the score of these alignments was reported (identity score and e-value) thus providing valuable indications of the cluster similarity with the reported genes (Table 4). Congruously, Table 4 reports clusters whose e-value was < 0.05 and showing a threshold of ± 2.000 log2fold change. The complete list of DEGs belonging to these pathways is reported in Supplementary file 9 (Table S2 A, B, C, D, E, F, G).

**Table 4:** Main processes affected by cadmium treatment

Cluster	Annotation	Gene identifier	Log_2_FoldChange	Identity %	e-value
*Phytohormone action*					
14,691.43609	nine-cis-epoxycarotenoid dioxygenase	AT1G78390.1	-3.2009	69	1.18e-179
14,691.50813	Abscisic acid 8'-hydroxylase 1-related	AT4G19230.1	2.0026	72	0
14,691.49474	Indole-3-pyruvate monooxygenase YUCCA2-related	AT5G25620.2	-2.6551	60	2.7e-74
14,691.13200	auxin efflux carrier component 9 (PIN)	AT5G57090.1	6,7678	53	1.50e-52
14,691.11710	1-Aminocyclopropane-1-carboxylate oxidase 51	AT1G77330.1	3.3103	63	9.05e-115
14,691.10785	ETHYLENE-INSENSITIVE 2 (EIN2)	AT5G03280.1	1.9616	54	3.91e-46
14,691.11833	gibberellin 20 oxidase 2	AT5G51810.1	2.9104	60	6.4e-162
14,691.10408	ROTUNDIFOLIA like 8	AT2G39705.1	7.0488	68	1.12e-09
14,691.12312	ROTUNDIFOLIA like 15	AT1G68825.1	6.1578	69	4.21e-10
15,957.0	CLAVATA3/ESR-RELATED 25	AT3G28455.1	6.0686	83	1.21e-4
14,691.82632	C-TERMINALLY ENCODED PEPTIDE 1	AT5G66815.1	9.4341	74	4.30e-04
*Transcription factors*					
14,691.27999	Ethylene-Responsive Transcription Factor CRF5-Related	AT2G20880.1	2.4027	92	3.47e-35
14,691.77282	Ethylene-Responsive Transcription Factor ERF003	AT5G25190.1	7.1758	70	1.54e-12
15,337.0	AP2-Like Ethylene-Responsive Transcription Factor AIL1	AT1G72570.1	7.117	87	5.02e-117
14,691.5263	Ethylene-Responsive Transcription Factor ERF071-Related	AT2G47520.1	5.4749	78	1.22e-12
14,691.17960	Ethylene-Responsive Transcription Factor CRF1-Related	AT3G14230.1	3.9041	96	4.31e-11
14,691.397	octadecanoid-responsive Arabidopsis AP2/ERF 59	AT1G06160.1	5.2872	61	2.22e-19
14,691.11372	related to AP2 11	AT5G19790.1	3.196	90	2.84e-14
14,691.28900	ERF domain protein 11	AT1G28370.1	2.4679	74	1.49e-03
14,691.32159	DNA-binding superfamily protein member of the ERF (ethylene response factor) subfamily	AT1G15360.1	-2.39	93	2.47e-12
14,691.82651	bZIP transcription factor family protein	AT1G68640.1	2.4872	63	3.05e-101
14,691.13800	Basic-leucine zipper (bZIP) transcription factor family protein	AT2G42380.2	3.7627	72	2.05e-42
19,280.1	WRKY DNA-binding protein 31	AT4G22070.1	6.1847	72	4.18e-63
14,691.7381	WRKY DNA-binding protein 9	AT1G68150.1	5.7632	63	4.79e-42
14,691.78281	WRKY DNA-binding protein 33	AT2G38470.1	5.4276	84	6.74e-29
32,613.0	WRKY DNA-binding protein 46	AT2G46400.1	-2.1896	70	5.80e-14
14,691.59464	WRKY family transcription factor	AT2G44745.1	-4.8279	77	4.73e-60
14,691.7255	WRKY DNA-binding protein 50	AT5G26170.1	4.2467	72	1.92e-25
14,691.57513	Transcription factor BHLH66-Related	AT4G30980.1	2.4623	98	3.22e-24
14,691.57184	Helix-loop-helix DNA-binding domain (HLH)	AT4G05170.1	-2.2109	83	1.35e-21
14,691.73182	Transcription factor BHLH71-RELATED	AT1G22490.1	7.0841	82	3.30e-15
14,691.61708	MYB domain PROTEIN 11-RELATED	AT2G47460.1	-2.8152	85	2.61e-15
14,691.15951	myb domain protein 24	AT5G40350.1	5.4902	82	3.71e-55
14,691.40443	Transcription factor RAX2	AT2G36890.1	-2.2491	78	6.01e-21
*Nutrient uptake*					
14,691.17907	nitrate transporter 2.5	AT1G12940.1	3.835	68	0
14,691.36103	nitrate reductase 1	AT1G77760.1	4.0674	61	8.24e-145
14,691.51441	phosphate transporter 1.7	AT3G54700.1	2.1772	71	1.34e-81
14,691.45899	phosphate transporter 1.4	AT2G38940.1	2.0997	76	0
14,691.23657	vacuolar iron transporter 1	AT2G01770.1	-2.4401	71	3.72e-58
*Cell wall*					
20,197.0	pectin methylesterase 61	AT3G59010.1	5.3189	65	3.93e-65
14,691.66836	Plant invertase/pectin methylesterase inhibitor superfamily	AT5G09760.1	4.3545	62	1.03e-108
14,691.14990	cinnamoyl coa reductase 1	AT2G33570.1	3.4503	64	2.51e-149
14,691.6397	laccase-8	AT3G09220.1	3,9852	42	2.24e-74
14,691.27253	membrane-associated progesterone binding protein 3	AT3G48890.1	2.2593	84	2.72e-16
14,691.76733	alpha expansin 11	AT1G20190.1	6.7503	73	6.73e-132
14,691.8155	EG45-LIKE domain containing protein 1 -related	AT4G30380.1	7.2163	61	3.44e-36
*Polyamine metabolism*					
14,691.48898	Agmatine deiminase	AT5G08170.1	-3.3113	72	1.82e-49
14,691.6139	Putative lysine decarboxylase family protein	AT2G28305.1	4.1682	82	1.93e-115
*ROS scavenging*					
14,691.12641	NADPH oxidase 1	AT1G09090.1	7.1387	50	5.55e-4
14,691.9595	Superoxide dismutase	AT1G08830.2	6.7735	55	2.49e-46
14,691.24413	Ascorbate peroxidase 3	AT4G35000.1	3.4836	84	7.24e-59
14,691.9539	Glutathione S-transferase	AT1G78340.1	9.5248	52	9.61e-66
10,504.0	Catalase	AT1G07890.6	6.3794	39	3.30e-4
14,691.81808	Glutaredoxin-C5	AT5G14070.1	7.2257	55	4.69e-35
*Heavy metal transporters*					
14,691.35424	Cadmium / zinc-transporting ATPase HMA3	AT4G30120.1	-3.7045	55	2.90e-42
27,204.0	Metal transporter Nramp1	AT1G80830.1	3.6765	57	2.06e-70

Regarding the “Phytohormone” category, nine-cis-epoxycarotenoid dioxygenase 9 catalyzing the first step of abscisic-acid (ABA) biosynthesis from carotenoids is down regulated in cadmium treated giant reed roots (Cd_R vs Cd_L*) (Table 4). Moreover, abscisic acid 8’-hydroxylase 1 – related involved in the first step of the oxidative degradation of ( +)-ABA was up-regulated in the Cd_R vs Cd_L* comparison, suggesting that cadmium treatment suppresses ABA synthesis and promotes its degradation in giant reed root thus quenching the signal cascade induced by this hormone. Indole-3-pyruvate monooxygenase YUCCA2-related encoding an enzyme involved in auxin biosynthesis and converting the indole-3-pyruvic acid (IPA) to indole-3-acetic acid (IAA) resulted up regulated in the cadmium treated roots with respect to treated leaf (Cd_R vs Cd_L*). Similarly, the gene encoding 1-aminocyclopropane-1-carboxylate oxidase (ACO) encoding an enzyme synthesizing the plant hormone ethylene was up-regulated in giant reed roots treated with cadmium. Gibberellin 20 oxidase 2, encoding a key oxidase enzyme in the biosynthesis of gibberellins, considered potent signaling molecules to enhance growth and developmental processes under abiotic stress conditions [52], was also found up-regulated in the Cd_R vs Cd_L* comparison. Signaling peptides, also known as peptide hormones, along with classical phytohormones, are involved in plant intracellular signaling. C-terminally encoded peptide (CEP), two genes encoding ROTUNDIFOLIA like proteins, and a transcript encoding CLAVATA3/ESR-RELATED 25 were among the sharply up-regulated genes in the cadmium treated root (Cd_R vs Cd_L*).

As concerns the “Transcription factors” category, several transcripts encoding Ethylene-Responsive Transcription (ERT) factors which have been reported to be involved in the ethylene signaling transduction pathway in plant abiotic stress response [53] were found sharply up regulated in the Cd_R vs Cd_L*. Therefore, the results indicate that ethylene-triggered signal cascade plays a crucial role in giant reed root under cadmium treatment. Interestingly, the WRKY gene family is also over-represented in the “Transcription factor” category (Table 4) and in particular WRKY9, that is involved in increased root suberin deposition [54], WRKY33 that controls the apoplastic barrier formation in roots [55] and WRKY50 positively regulating resistance against necrotrophic pathogens [56] were found up-regulated in cadmium treated giant reed roots (Cd_R vs Cd_L*). Conversely, WRKY46 that seems to be involved in hypersensitivity to drought and salt stress [57] was down regulated (Table 4).

The analysis of the “Cell wall” category reveals that transcripts encoding alpha expansin 11, that causes loosening and extension of plant cell walls by disrupting non-covalent bonding between cellulose microfibrils and matrix glucans [58], were up-regulated in the Cd_R vs Cd_L* comparison. The genes encoding cinnamoyl Coa reductase, laccase and membrane-associated progesterone binding protein 3 involved in lignin biosynthesis were strongly induced in cadmium treated roots. Moreover, pectin methylesterase 61 and invertase/pectin methylesterase inhibitor gene have been found up-regulated in Cd-treated root thus confirming the overall results related to “Cell wall” category that this cellular district is target of deep remodeling under cadmium stress [59]. Regarding the “Nutrient uptake” category, transcripts related with nitrogen metabolism, such as nitrate reductase and high affinity nitrate transporter 2.5 were upregulated by cadmium treatment in giant reed roots, being they involved in nitrogen assimilation and uptake, respectively. Furthermore, phosphate transporter encoding genes involved in phosphate uptake at plasma membrane level were also upregulated whereas vacuolar iron transporter required for iron sequestration into vacuoles was downregulated in cadmium treated roots. Overall, these results support that cadmium treatment induced an imbalance of the main nutrient levels which can cause disturbances in plant growth. Polyamines (PAs) are ubiquitous low molecular weight aliphatic cations that are present in all organisms; the major PAs in plants are putrescine, spermidine, and spermine, and to a lesser extent, cadaverine. Cadaverine, a structurally different diamine, has an independent biosynthetic pathway as it is synthesized from lysine by lysine decarboxylase (LDC) [60]. Agmatine deiminase catalyzes the hydrolysis of agmatine into N-carbamoylputrescine in the arginine decarboxylase (ADC) pathway of putrescine biosynthesis. In the “Polyamine metabolism” category, lysine decarboxylase gene was upregulated whereas agmatine deiminase was downregulated in cadmium treated roots, suggesting that under heavy metal stress, the pathway leading to cadaverine is preferentially undertaken in giant reed root.

The amount of cadmium inside the root cells depends on the balance between metal uptake, normally determined by general metal transporters belonging to the NRAMP family, and metal extrusion. The heavy metal ATPase 3 (HMA3) transporters translocate cadmium both outside the cell and into the vacuole [61, 62]. The analysis of “Metal transporter” group revealed that transcript encoding NRAMP1 transporter was upregulated whilst HMA3 transporter encoding gene were downregulated in the cadmium treated root thus indicating that the mechanisms to avoid cadmium uptake and stay are not implemented in our conditions.

As concerns the ROS scavenging regulatory mechanisms, the analysis of Cd_R vs Cd_L* revealed that superoxide dismutase (SOD), ascorbate peroxidase (APX), catalase and NADPH oxidase 1 encoding genes are all up-regulated suggesting that H_2_O_2_ could be the main ROS the *A. donax* roots have to cope with under cadmium treatment. Moreover, many transcripts encoding glutathione transferases (GSTs) are also up-regulated (Table 4) concordantly with their role in abiotic stress relief [63, 64]. Moreover, transcripts encoding glutaredoxin-C5, small redox enzyme of approximately one hundred amino-acid residues involved in the reduction of specific disulfides such as glutathionylated proteins [65] were found strongly induced in cadmium treated roots. Finally, the “Protein biosynthesis” category encompasses a plethora of genes involved in ribosome constitution (ribosomal proteins) and DNA-directed RNA polymerase II subunit that resulted up regulated indicating that this pathway is sharply activated by cadmium treatment in giant reed roots (Table S2C).

### Analysis of the *A. donax* response to salt and cadmium treatments

**Fig. 5:**
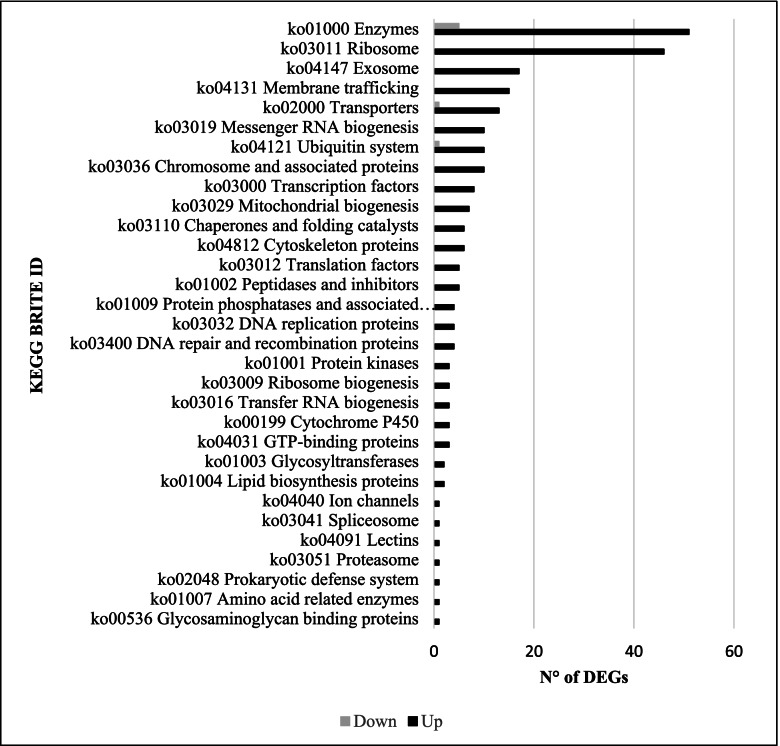
KO classification of DEGs found in common between salt and cadmium stress in *A.donax* ecotypes

The availability of transcriptomic data enabled the identification of genes that are implicated in *A. donax* response to both salt [31, 32] and cadmium stress. In details, a DEG list containing deregulated genes in both stress conditions was obtained by using the KEGG ORTHOLOGY database (see Materials and Methods). The KEGG ORTHOLOGY database represents a reference resource for gene and protein annotation and allowed to overcome the species-specific annotation of other databases potentially leading to bias in the analysis [66]. Figure 5 reports the classification of common DEGs and their numerical distribution. Based on this analysis 162 DEGs were successfully classified and most of them (156) were up-regulated in both stress conditions whereas only 6 DEGs were down-regulated. In particular, the most abundant KO category was (ko01000) Enzymes (51 up-regulated and 4 down regulated DEGs), followed by (ko03011) Ribosome (46 up-regulated DEGs), (ko04147) Exosome (17 up-regulated DEGs), (ko04131) Membrane trafficking (15 up-regulated DEGs) and (ko02000) Transporters (13 up-regulated and 1 down-regulated DEGs) (Fig. 5). Among the DEGs included in the “Transcription factor” category AP2-like factor and EREBP-like factor were found up-regulated, along with amino-cyclopropane carboxylate oxidase (ACO) confirming the crucial role of ethylene in giant reed abiotic stress response. Moreover, the “Ribosome” category (46 up-regulated DEGs) contains an elevated number of genes encoding ribosomal proteins indicating that “Protein biosynthesis” is an extremely involved pathway in both conditions. The complete list containing the KO description of each DEG involved in both salt and cadmium stress conditions is in Supplementary file 10: Table S3.

## Discussion

Anthropogenic activities such as metal industries, mining application of pesticides and fertilizers led to dangerous Cd accumulation in arable soil worldwide [61]. The high solubility of Cd in the soil associated with the plant capability to absorb it represents a threat to humans as final consumers of putatively contaminated fruit and vegetables. The strategy to allocate cadmium contaminated soil to bioenergy crop might turn out to be successful as it potentially solves the increasing demand of sustanible energy sources which can be achieved without impairing the quote of agricultural lands. *Arundo donax* L. is considered tolerant to several abiotic stress and recently, due to its ability to accumulate high concentration of HMs [24, 25] has been proposed as a potential phytoremediation species. In order to elucidate the response of giant reed to cadmium, in this work we sequenced and de novo assembled the *A. donax* L. leaf and root transcriptome after a prolonged soil treatment with 4 mg/Kg cadmium nitrate. Cadmium concentrations in uncontaminated soil usually is 0.5 mg/Kg and cadmium contents above 3 mg/kg are generally thought to indicate contaminated soil [37]. Based on the measurement of trace cadmium concentration under non-phytotoxic cadmium treatment, Bonanno (2012) [67] showed that giant reed roots act as main accumulation centre (root to shoot ratio is 6.6:1), whereas stem functions as transition centre given the involvement in ion translocation from root to leaves. Taken into account these observations which preliminarily indicated that root is the frontline in encountering potentially toxic cadmium levels, we focused our analysis on a Cd_R vs Cd_L* comparison originated by subtracting the DEGs belonging to the CK_R vs CK_L comparison (root specific DEGs under control conditions) to the Cd_R vs Cd_L comparison (root specific DEGs under cadmium treatment) thus obtaining a pool of DEGs specifically up or down-regulated (compared to leaf DEGs) in treated roots. Based on differentially gene expression data, the up regulation of the NRAMP1 transporter, a crucial group of transmembrane protein involved in the uptake of a broad range of ions (Mn, Zn, Cu, Fe, Ni, Co and Cd), suggests that giant reed roots reinforce their ability to adsorb ions in the presence of cadmium which interferes with the essential mineral supply [61]. Moreover, the down regulation of HMA3 transporter encoding gene, that regulates cadmium efflux out the citosol towards the vacuole or outside the cell [51], indicates that the mechanisms to avoid cadmium residence in root cells are not implemented in our conditions.

Once in the citosol, Cd has to be detoxified in order to avoid the onset of cellular damage. It has been shown that the major mechanism of cadmium detoxification is based on phytochelatins, a family of cysteine-rich oligopeptides synthesized from glutathione (GSH) by the enzyme phytochelatin synthase (PCS), that chelates the HMs to form complexes readily stored in the vacuoles [68]. Recently, three giant reed PCS genes have been characterized both in the species of provenance and in transgenic model organisms [34]. These genes, namely *Ad*PCS1-3, are expressed in all organs (root, rhizome, node, internode, leaf sheath and blade) in normal condition. In response to 500 μM CdSO_4_ treatment, all the PCS genes showed an increase of expression in roots whereas any change in leaf gene expression was observed [34]. Although these results clearly indicate that *A. donax* genome contains at least three PCS genes constitutively expressed in all plant organs and whose expression is induced in roots under cadmium treatment, PCSs were found among the DEGs (data not shown) suggesting that the detoxification process through metal chelation was not triggered in our conditions.

After the stress perception, phytohormones are the chemical messengers that play a pivotal role in the induction and regulation of diverse signal transduction pathways in response to cadmium stress [69]. Higher endogenous abscisic acid (ABA) concentration are reported to mitigate the damaging effects of Cd stress by promoting root-to-shoot Cd translocation through the apoplast and more Cd accumulation in the shoots [70]. Our results suggested that abscisic acid biosynthesis is inhibited whereas the main gene involved in ABA degradation is up-regulated in giant reed Cd-treated roots (Table 4), probably causing Cd to be detained in the roots. Auxin is recognized as a crucial phytohormone in regulating every aspect of plant root development during normal and stress conditions [71]. In *Arabidopsis thaliana*, significantly enhanced expression of auxin biosynthesis gene YUCCA6 was observed upon Cd treatment [72]. Moreover, reduced transcript levels of auxin efflux carrier pin-formed 1 (PIN1) were detected under Cd exposure in the post-embryonic roots decreasing auxin transport in the root apex thus altering auxin homeostasis [72]. In our study, both auxin biosynthesis and transport transcript (YUCCA2 and PIN) were found among the up-regulated genes in Cd-treated giant reed roots suggesting that roots attempt not to modify auxin homeostasis preventing the alteration of root architecture as observed in tobacco [73]. Furthermore, our results strongly indicate that ethylene biosynthesis and the downstream signaling cascade are up regulated in Cd-treated roots (Table 4). Similarly, the enhanced transcript levels of 1-aminocyclopropane-1-carboxylate (ACO) and ethylene signaling genes (EIN) were observed under As applicationin the rice roots [74], and transcript level accumulation of ethylene receptor 2 (ETR2) gene was also observed under Cd application in *Arabidopsis thaliana* generally suggesting the occurrence of Cd-mediated induction of ethylene production and signaling [75].

Several studies indicated that ethylene modulates the ROS machinery by regulating the activities of both ROS producing and scavenging enzymes, this being considered the crucial reaction following HM stress in plants [76]. Ethylene stimulates the production of ROS by activating nicotinamide adenine dinucleotide phosphate (NADPH) oxidases under HM toxicity [77] and also modulates antioxidant defense systems in plants exposed to HM stress. NADPH oxidase 1 transcript was among the up regulated genes in Cd-treated roots and the activation of catalase, SOD, APX and GST expression (Table 4) is probaly an attempt to overcome the Cd-induced oxidative stress. This result assumes an important significance since in several species it has been reported that the activation of ROS scavenging machinery is achieved in ethylene insensitive mutants [69] thus suggesting that this type of response could be specific of *A. donax* roots. However, we cannot exclude that the induction of YUCCA2 involved in auxin biosynthesis might be responsible of the regulation of reactive oxygen species (ROS) homeostasis as showed in transgenic potato over-expressing YUCCA6 gene [78]. Oxidative stress can induce protein S-glutathionylation modulating protein function and to prevent irreversible oxidation of protein thiols [79]. High levels of oxidized glutathione (GSSG) can be sufficient to trigger protein S-glutathionylation by a thiol–disulphide exchange reaction between a protein thiol and GSSG. Interestingly, transcript encoding glutaredoxin-C5 is sharply up regulated in giant reed roots indicating that an active reactivation of protein function via the reduction of the cysteine sulphydrilic groups might occurr.

Within the “Phytohormones” category, two genes encoding ROTUNDIFOLIA like proteins, which are small polypeptides acting as a regulatory molecules coordinating cellular responses involved in different aspects of cell differentiation, growth and development, were found sharply up regulated in the Cd_R vs Cd_L* comparison. Their role is uncertain but probably they act by restricting polar cell proliferation in lateral organs and coordinating socket cell recruitment and differentiation at trichome sites [80]. A transcript encoding CLAVATA3/ESR-RELATED 25 is among the sharply up-regulated genes in the cadmium treated root (Table 4), CLAVATA3/ESR represents a group of plant proteins acting as extracellular signal peptides involved in cell-to-cell communication in concert with different receptors in a range of processes during plant development [81].

A transcript encoding C-terminally encoded peptide (CEP) was strongly up-regulated in cadmium treated roots. CEP is a 15-amino acid post-translationally peptide which plays a pivotal role in lateral root formation and nodulation and its overexpression in *Medicago* results in the inhibition of lateral root formation and enhancement in nodule formation. Besides the role in root architecture, CEP genes also play a role in nitrate assimilation under N starvation conditions and provoke the up regulation of the nitrate transporter gene NRT2.1 in roots specifically when nitrate is present in the rhizosphere [82]. Nitrate transporter are involved in the constitutive high-affinity transport system (cHATS) under low nitrate conditions. The principal role of this cHATS is to enable roots previously deprived of nitrate to absorb this ion and initiate induction of nitrate-inducible genes [83]. Cd has been reported to inhibit NO_3_^−^ uptake in several plant species, under normal and high nitrate supply [84]. Therefore, it is possible that nitrate uptake is inhibited under Cd stress in giant reed roots mimicking low-nitrate conditions. Interestingly, the analysis of “Nutrient uptake” category revealed that nitrate transporter 2.5 genes are strongly up-regulated (Table 4) suggesting that mechanisms to enhance nitrogen supply are implemented in *A. donax* cadmium treated roots. Moreover, nitrate reductase encoding gene, involved in nitrate assimilation by reducing nitrate to nitrite, is up-regulated in cadmium treated samples probably to provide for more enzyme molecules that are supposed to be inhibited by cadmium [85]. Under cadmium stress a decrease of mineral nutrient concentrations in plant leaf, such as Fe and P, has been observed and represents the key reason for the restraint of leaf photosynthesis [61]. In our work, transcript encoding the plasma membrane phosphate transporter are up-regulated in Cd-treated roots probably to face the unavailability of soluble phosphate sequestered in the soil as cadmium phosphate [86].

The plant cell has a variety of mechanisms tools to avoid Cd stress. Mainly, cell wall remodeling might prevent Cd from entering and damaging the protoplast [87]. At primary cell wall, pectin, which contains most of the negative charges, can immobilize Cd very effectively. Furthermore, secondary cell wall lignification can serve to create a barrier to prevent cadmium entry. The utilization of different antibodies detecting methylated and demethylated forms of pectin in cadmium stressed *Linum usitatissimum* hypocotyls has led to the identification of low-methylated pectin, which is particularly able to bind Cd ions due to the presence of free carboxyl groups in the outer side of the primary cell wall. The increased low-methylated pectin form occurs along with the co-localized upregulation of pectin methylesterase (PME) activity. Instead, a higher amount of methylated pectin was detected within the inner side of the primary cell wall which was hypothesized to have an impermeable function aimed to keep the cytosolic Cd away from the cell [88]. In this perspective, the up regulation of both PME and PME inhibitor encoding genes might serve to finely regulate Cd sequestering at primary cell wall in giant cane root. At secondary cell wall level, lignification makes the cell wall less penetrable thus creating an effective barrier against the entry of Cd [89]. The induction of the lignification process appeared as a key process useful to discriminate Cd-sensitive and -tolerant plants [90, 91]. The discovery of the upregulation of several WRKY transcription factors involved in cell wall lignification as well as the induction of cinnamoyl CoA reductase, laccase and membrane-associated progesterone binding protein 3, all of them involved in lignin biosynthesis (Table 4), clearly indicate that giant cane roots respond to cadmium treatment by avoiding its entrance into the cell.

## Conclusions

The global analysis of our findings suggests that prolonged cadmium exposure stimulated a clear response at both morpho-physiological and transcriptomic levels. Hence, cadmium treated plants showed significantly reduced main stem height, biomass dry weight and the net photosynthesis efficiency. The quality of transcriptome sequencing and assembly was elevated and led to the identification of crucial metabolic pathways and to decipher the *A. donax* response to cadmium stress. Three main factors have to be taken in strong consideration in this concluding remarks: a) the used cadmium concentration (4 mg/Kg), slightly higher than allowed; b) the induction of lignification process clearly suggested by transcriptome analysis; c) the lack of phytochelatin transcripts among the DEGs. In our opinion, all these issues indicate that the induced stress condition can be sensed as “mild stress”. The low number of DEGs within the CK_R vs Cd_L and CK_L vs_Cd_L comparisons seems to be in line with this hypothesis. The undertaken strategy was to analyse the Cd_R vs Cd_L* comparison and it led us to focus on the main patterns involved in the Cd-treated giant cane root, it being the interface between plant and soil. Our results suggest that ethylene biosynthesis and signaling are strongly activated. In this respect, the identification of several genes differently regulated in both salt and cadmium conditions, such as genes involved in ethylene biosynthesis and signal transduction, outlines those metabolic pathways and biochemical reactions as useful markers of abiotic stress in giant reed. Finally, the finding of DEGs encoding several small peptides functioning as messenger molecules between root and shoot in order to communicate the stressful status to the upper part of the plants (CEP, ROTUNDIFOLIA), the induction of the ROS scavenging machinery, and, above all, the remodelling of plant cell wall confirm the tolerance of giant cane towards cadmium stress and strongly support its cultivation in cadmium contaminated soils in a perspective to save agricultural soil for food and feed crops.

## Supplementary Information

**Additional file 1: Figure S1.** Picture of giant reed plants at sampling date (July 28^th^, 2020).

**Additional file 2: Figure S2.** Effect of cadmium treatment on morpho-biometric and physiological parameters of *A. donax* G10 ecotype. A) Main stem height per pot. B) Biomass dry weight. C) Net photosynthesis efficiency.

**Additional file 3: Figure S3.** Flowchart of sequencing and *de novo* assembly of *A.donax* leaf and root transcriptome under cadmium treatment.

**Additional file 4: Figure S4.** Length distribution of transcripts and unigenes.

**Additional file 5: Table S1.** Complete DEG list of (Cd_R vs CK_R) and (Cd_L vs CK_L) comparisons.

**Additional file 6: Figure S5.** Three-dimension PCA for RNAseq correlation. Principal Component coordinates were calculated using sample read counts. **Figure S6** - Hierarchical clustering map for differential expression genes.

**Additional file 7: Figure S7.** Validation of *A. donax* DEGs by Real Time qRT-PCR.

**Additional file 8: Figure S8.** KOG functional classification.

**Additional file 9: Tables S2.** (A, B, C, D, E, F, G). List of significant DEGs of the Cd_R vs Cd_L* comparison.

**Additional file10: Table S3.** KO description of DEGs found in common between salt and cadmium treatment.

## Data Availability

The *Arundo donax* leaf and root transcriptome was submitted to NCBI (https://www.ncbi.nlm.nih.gov/geo/) accession number GSE195580.

## Abbreviations

ABA: Abscisic acid
AUX/IAA: Auxin/indole acetic acid
BP: Biological process
MF: Molecular function
DEGs: Differentially expressed genes
GHG: Greenhouse gases emission
GO: Gene ontology
KEGG: Kyoto encyclopedia of genes and genomes
KO: Ortholog database
KOG/COG: Clusters of orthologous groups of proteins
NCBI: National center for biotechnology information
Nr: NCBI non-redundant protein sequences
Nt: NCBI non-redundant nucleotide sequences
Pfam: Protein family
qRT-PCR: Quantitative real-time PCR
RIN: RNA integrity number
ROS: Reactive oxygen species
TFs: Transcription factors

## References

[1] REN21 (2016). Renewables 2016 Global Status Report.

[2] Muench S, Guenther E (2013). A systematic review of bioenergy life cycle assessments. Appl Energy..

[3] Roos A, Ahlgren S (2018). Consequential life cycle assessment of bioenergy systems–a literature review. J Clean Prod..

[4] Lewandowski I, Clifton-Brown J, Trindade LM, Van der Linden GC, Schwarz KU, Müller-Sämann K, Anisimov A, Chen CL, Dolstra O, Donnison IS, Farrar K (2016). Progress on optimizing miscanthus biomass production for the European bioeconomy: Results of the EU FP7 project OPTIMISC. Front Plant Sci..

[5] United Nations, Department of Economic and Social Affairs, Population Division (2019). World Population Prospects 2019: Highlights (ST/ESA/SER.A/423).

[6] Rivera A, Bravo C, Buob G. Climate change and land Ice. Int Encyclopedia Geogr: People Earth Environ Technol. 2017:1–15.

[7] Edelstein M, Ben-Hur M (2018). Heavy metals and metalloids: Sources, risks and strategies to reduce their accumulation in horticultural crops. Sci Hortic..

[8] Tchounwou PB, Yedjou CG, Patlolla AK, Sutton DJ. Heavy metal toxicity and the environment. Exp Suppl. 2012:133–64. 10.1007/978-3-7643-8340-4_6 PMC4144270 22945569

[9] Mahar A, Wang P, Ali A, Awasthi MK, Lahori AH, Wang Q, Li R, Zhang Z (2016). Challenges and opportunities in the phytoremediation of heavy metals contaminated soils: a review. Ecotoxicol Environ Saf..

[10] Pan J, Plant JA, Voulvoulis N, Oates CJ, Ihlenfeld C (2010). Cadmium levels in Europe: implications for human health. Environ Geochem Health..

[11] Sidhu GP, Bali AS, Bhardwaj R, Hasanuzzaman M, MNV P, Nahar K (2019). Role of organic acids in mitigating cadmium toxicity in plants.

[12] Gall JE, Boyd RS, Rajakaruna N (2015). Transfer of heavy metals through terrestrial food webs: a review. Environ Monit Assess..

[13] Fan W, Liu C, Cao B, Ma S, Hu J, Xiang Z, Zhao A (2021). A meta-analysis of transcriptomic profiles reveals molecular pathways response to cadmium stress of Gramineae. Ecotoxicol Environ Saf..

[14] Nevo Y, Nelson N (2006). The NRAMP family of metal-ion transporters. Biochim Biophys Acta - Mol Cell Res..

[15] Stohs SJ, Bagchi D (1995). Oxidative mechanisms in the toxicity of metal ions. Free Radic Biol Med..

[16] Yadav SK (2010). Heavy metals toxicity in plants: an overview on the role of glutathione and phytochelatins in heavy metal stress tolerance of plants. S Afr J Bot..

[17] Sharma SS, Dietz KJ (2009). The relationship between metal toxicity and cellular redox imbalance. Trends Plant Sci..

[18] DalCorso G, Manara A, Furini A (2013). An overview of heavy metal challenge in plants: from roots to shoots. Metallomics..

[19] Lewandowski I, Scurlock JM, Lindvall E, Christou M (2003). The development and current status of perennial rhizomatous grasses as energy crops in the US and Europe. Biomass Bioenergy..

[20] Angelini LG, Ceccarini L (2009). o Di Nasso NN, Bonari E. Comparison of Arundo donax L. and Miscanthus x giganteus in a long-term field experiment in Central Italy: Analysis of productive characteristics and energy balance. Biomass Bioenergy..

[21] Bajguz A, Hayat S (2009). Effects of brassinosteroids on the plant responses to environmental stresses. Plant Physiol Biochem..

[22] Zhang JJ, Lu YC, Zhang SH, Lu FF, Yang H (2016). Identification of transcriptome involved in atrazine detoxification and degradation in alfalfa (Medicago sativa) exposed to realistic environmental contamination. Ecotoxicol Environ Saf..

[23] Fernando AL, Barbosa B, Costa J, Papazoglou EG, MNV P (2016). Giant reed (Arundo donax L.): A multipurpose crop bridging phytoremediation with sustainable bioeconomy. Bioremediation and Bioeconomy.

[24] Papazoglou EG, Serelis KG, Bouranis DL (2007). Impact of high cadmium and nickel soil concentration on selected physiological parameters of Arundo donax L. Eur J Soil Sci..

[25] Mirza N, Pervez A, Mahmood Q, Shah MM, Shafqat MN (2011). Ecological restoration of arsenic contaminated soil by Arundo donax L. Ecol Eng..

[26] Cheng H, Jiang ZY, Liu Y, Ye ZH, Wu ML, Sun CC, Sun FL, Fei J, Wang YS (2014). Metal (Pb, Zn and Cu) uptake and tolerance by mangroves in relation to root anatomy and lignification/suberization. Tree Physiol..

[27] Sablok G, Fu Y, Bobbio V, Laura M, Rotino GL, Bagnaresi P, Allavena A, Velikova V, Viola R, Loreto F, Li M (2014). Fuelling genetic and metabolic exploration of C 3 bioenergy crops through the first reference transcriptome of A rundo donax L. Plant Biotechnol J..

[28] Barrero RA, Guerrero FD, Moolhuijzen P, Goolsby JA, Tidwell J, Bellgard SE, Bellgard MI (2015). Shoot transcriptome of the giant reed. Arundo donax. Data in Brief..

[29] Evangelistella C, Valentini A, Ludovisi R, Firrincieli A, Fabbrini F, Scalabrin S, Cattonaro F, Morgante M, Mugnozza GS, Keurentjes JJ, Harfouche A (2017). De novo assembly, functional annotation, and analysis of the giant reed (Arundo donax L.) leaf transcriptome provide tools for the development of a biofuel feedstock. Biotechnol Biofuels..

[30] Fu Y, Poli M, Sablok G, Wang B, Liang Y, La Porta N, Velikova V, Loreto F, Li M, Varotto C (2016). Dissection of early transcriptional responses to water stress in Arundo donax L. by unigene-based RNA-seq. Biotechnol Biofuels..

[31] Sicilia A, Testa G, Santoro DF, Cosentino SL, Lo Piero AR (2019). RNASeq analysis of giant cane reveals the leaf transcriptome dynamics under long-term salt stress. BMC Plant Biol..

[32] Sicilia A, Santoro DF, Testa G, Cosentino SL, Lo Piero AR (2020). Phytochemistry.

[33] Shaheen S, Ahmad R, Mahmood Q, Mubarak H, Mirza N, Hayat MT (2018). Physiology and selected genes expression under cadmium stress in Arundo donax L. Int J Phytoremediation..

[34] Li M, Stragliati L, Bellini E, Ricci A, Saba A (2019). Sanità di Toppi L, Varotto C. Evolution and functional differentiation of recently diverged phytochelatin synthase genes from Arundo donax L. J Exp Bot..

[35] Cosentino SL, Copani V, D’Agosta GM, Sanzone E, Mantineo M (2006). First results on evaluation of Arundo donax L. clones collected in Southern Italy. Ind Crops Prod..

[36] Commission E (1986). Protection of the Environment, and in particular of the soil, when sewage sludge is used in agriculture. Off J Eur Communities..

[37] Akbar KF, Hale WH, Headley AD, Athar M (2006). Heavy metal contamination of roadside soils of Northern England. Soil Water Res..

[38] Grabherr MG, Haas BJ, Yassour M, Levin JZ, Thompson DA, Amit I, Adiconis X, Fan L, Raychowdhury R, Zeng Q, Chen Z (2011). Trinity: reconstructing a full-length transcriptome without a genome from RNA-Seq data. Nat Biotechnol..

[39] Davidson NM, Oshlack A (2014). Corset: enabling differential gene expression analysis for de novo assembled transcriptomes. Genome Biol..

[40] Simão FA, Waterhouse RM, Ioannidis P, Kriventseva EV, Zdobnov EM (2015). BUSCO: assessing genome assembly and annotation completeness with single-copy orthologs. Bioinformatics..

[41] Buchfink B, Xie C, Huson DH (2015). Fast and sensitive protein alignment using DIAMOND. Nat Methods..

[42] Finn RD, Clements J, Eddy SR (2011). HMMER web server: interactive sequence similarity searching. Nucleic Acids Res.

[43] Moriya Y, Itoh M, Okuda S, Yoshizawa AC, Kanehisa M (2007). KAAS: an automatic genome annotation and pathway reconstruction server. Nucleic Acids Res.

[44] Götz S, García-Gómez JM, Terol J, Williams TD, Nagaraj SH, Nueda MJ, Robles M, Talón M, Dopazo J, Conesa A (2008). High-throughput functional annotation and data mining with the Blast2GO suite. Nucleic Acids Res..

[45] Pérez-Rodríguez P, Riano-Pachon DM, Corrêa LG, Rensing SA, Kersten B, Mueller-Roeber B (2010). PlnTFDB: updated content and new features of the plant transcription factor database. Nucleic Acids Res.

[46] Jin HJ, Zhao JC, Wu L, Kim J, Yu J (2014). Cooperativity and equilibrium with FOXA1 define the androgen receptor transcriptional program. Nat Commun..

[47] Li B, Dewey CN (2011). RSEM: accurate transcript quantification from RNA-Seq data with or without a reference genome. BMC Bioinform..

[48] Love MI, Huber W, Anders S (2014). Moderated estimation of fold change and dispersion for RNA-seq data with DESeq2. Genome Biol..

[49] Guo J, Song J, Wang F, Zhang XS (2007). Genome-wide identification and expression analysis of rice cell cycle genes. Plant Mol Biol..

[50] Mao X, Cai T, Olyarchuk JG, Wei L (2005). Automated genome annotation and pathway identification using the KEGG Orthology (KO) as a controlled vocabulary. Bioinformatics..

[51] Thimm O, Bläsing O, Gibon Y, Nagel A, Meyer S, Krüger P, Selbig J, Müller LA, Rhee SY, Stitt M (2004). MAPMAN: a user‐driven tool to display genomics data sets onto diagrams of metabolic pathways and other biological processes. Plant J..

[52] Colebrook EH, Thomas SG, Phillips AL, Hedden P (2014). The role of gibberellin signalling in plant responses to abiotic stress. J Exp Biol..

[53] Nakano T, Suzuki K, Fujimura T, Shinshi H (2006). Genome-wide analysis of the ERF gene family in Arabidopsis and rice. Plant Physiol..

[54] Krishnamurthy P, Vishal B, Bhal A, Kumar PP (2021). WRKY9 transcription factor regulates cytochrome P450 genes CYP94B3 and CYP86B1, leading to increased root suberin and salt tolerance in Arabidopsis. Physiol Plant..

[55] Krishnamurthy P, Vishal B, Ho WJ, Lok FC, Lee FS, Kumar PP (2020). Regulation of a cytochrome P450 gene CYP94B1 by WRKY33 transcription factor controls apoplastic barrier formation in roots to confer salt tolerance. Plant Physiol..

[56] Hussain RM, Sheikh AH, Haider I, Quareshy M, Linthorst HJ (2018). Arabidopsis WRKY50 and TGA transcription factors synergistically activate expression of PR1. Front Plant Sci..

[57] Ding ZJ, Yan JY, Li CX, Li GX, Wu YR, Zheng SJ (2015). Transcription factor WRKY 46 modulates the development of Arabidopsis lateral roots in osmotic/salt stress conditions via regulation of ABA signaling and auxin homeostasis. Plant J..

[58] Wang T, Park YB, Caporini MA, Rosay M, Zhong L, Cosgrove DJ, Hong M (2013). Sensitivity-enhanced solid-state NMR detection of expansin’s target in plant cell walls. PNAS..

[59] Wormit A, Usadel B (2018). The multifaceted role of pectin methylesterase inhibitors (PMEIs). Int J Mol Sci..

[60] Rajpal C, Tomar PC (2020). Cadaverine: a Potent Modulator of Plants Against Abiotic Stresses. J Microbiol Biotechnol Food Sci..

[61] Nazar R, Iqbal N, Masood A, Khan MIR, Syeed S, Khan NA (2012). Cadmium Toxicity in Plants and Role of Mineral Nutrients in Its Alleviation. Am J Plant Sci..

[62] Mohabubul Haque AFM, Gohari G, El-Shehawi AM, Dutta AK, Elseehy MM, Kabir AH (2022). Genome-wide identification, characterization and expression profiles of heavy metal ATPase 3 (HMA3) in plants. J King Saud Univ - Sci..

[63] Lo Piero AR, Mercurio V, Puglisi I, Petrone G (2009). Gene isolation and expression analysis of two distinct sweet orange [Citrus sinensis L. (Osbeck)] tau-type glutathione transferases. Gene..

[64] Puglisi I, Lo Cicero L, Lo Piero AR (2013). The glutathione S-transferase gene superfamily: An in silico approach to study the post translational regulation. Biodegradation..

[65] Couturier J, Ströher E, Albetel AN, Roret T, Muthuramalingam M, Tarrago L (2011). Arabidopsis chloroplastic glutaredoxin C5 as a model to explore molecular determinants for iron-sulfur cluster binding into glutaredoxins. J Biol Chem..

[66] Kanehisa M, Sato Y, Kawashima M, Furumichi M, Tanabe M (2016). KEGG as a reference resource for gene and protein annotation. Nucleic Acids Res..

[67] Bonanno G (2012). Arundo donax as a potential biomonitor of trace element contamination in water and sediment. Ecotoxicol Environ Saf..

[68] Song WY, Mendoza-Cózatl DG, Lee Y, Schroeder JI, Ahn SN, Lee HS (2014). Phytochelatin-metal(loid) transport into vacuoles shows different substrate preferences in barley and Arabidopsis. Plant Cell Environ..

[69] Saini S, Kaur N, Pati PK (2021). Phytohormones: Key players in the modulation of heavy metal stress tolerance in plants. Ecotoxicol Environ Saf..

[70] Lu Q, Chen S, Li Y, Zheng F, He B, Gu M (2020). Exogenous abscisic acid (ABA) promotes cadmium (Cd) accumulation in Sedum alfredii Hance by regulating the expression of Cd stress response genes. Environ Sci Pollut Res..

[71] Saini S, Sharma I, Kaur N, Pati PK (2013). Auxin: A master regulator in plant root development. Plant Cell Rep..

[72] Fattorini L, Ronzan M, Piacentini D, Della Rovere F, De Virgilio C, Sofo A (2017). Cadmium and arsenic affect quiescent centre formation and maintenance in Arabidopsis thaliana post-embryonic roots disrupting auxin biosynthesis and transport. Environ Exp Bot..

[73] Luo Y, Wei Y, Sun S, Wang J, Wang W, Han D, Shao H, Jia H, Fu Y (2019). Selenium modulates the level of auxin to alleviate the toxicity of cadmium in tobacco. Int J Mol Sci..

[74] Huang TL, Nguyen QTT, Fu SF, Lin CY, Chen YC, Huang HJ (2012). Transcriptomic changes and signalling pathways induced by arsenic stress in rice roots. Plant Mol Biol..

[75] Schellingen K, Van Der Straeten D, Vandenbussche F, Prinsen E, Remans T, Vangronsveld J (2014). Cadmium-induced ethylene production and responses in Arabidopsis thaliana rely on ACS2 and ACS6 gene expression. BMC Plant Biol..

[76] Steffens B (2014). The role of ethylene and ROS in salinity, heavy metal, and flooding responses in rice. Front Plant Sci..

[77] Montero-Palmero MB, Martín-Barranco A, Escobar C, Hernández LE (2014). Early transcriptional responses to mercury: A role for ethylene in mercury-induced stress. New Phytol..

[78] Kim JI, Baek D, Park HC, Chun HJ, Oh DH, Lee MK (2013). Overexpression of arabidopsis YUCCA6 in potato results in high-auxin developmental phenotypes and enhance. Mol Plant..

[79] Dalle-Donne I, Rossi R, Colombo G, Giustarini D, Milzani A (2009). Protein S-glutathionylation: a regulatory device from bacteria to humans. Trends Biochem Sci..

[80] Valdivia ER, Chevalier D, Sampedro J, Taylor I, Niederhuth CE, Walker JC (2012). DVL genes play a role in the coordination of socket cell recruitment and differentiation. J Exp Bot..

[81] Strabala TJ, O’Donnell PJ, Smit AM, Ampomah-Dwamena C, Martin EJ, Netzler N (2006). Gain-of-function phenotypes of many CLAVATA3/ESR genes, including four new family members, correlate with tandem variations in the conserved CLAVATA3/ESR domain. Plant Physiol..

[82] Ohkubo Y, Tanaka M, Tabata R, Ogawa-Ohnishi M, Matsubayashi Y (2017). Shootto-root mobile polypeptides involved in systemic regulation of nitrogen acquisition. Nat Plants..

[83] Guan MY, Chen MX, Cao ZZ (2021). NRT2.1, a major contributor to cadmium uptake controlled by high-affinity nitrate transporters. Ecotoxicol Environ Saf.

[84] Guan MY, Fan SK, Fang XZ, Jin CW (2015). Modification of nitrate uptake pathway in plants affects the cadmium uptake by roots. Plant Signal Behav..

[85] Singh P, Singh I, Shah K (2019). Reduced activity of nitrate reductase under heavy metal cadmium stress in rice: an in silico answer. Front Plant Sci..

[86] Ruangcharus C, Kim SU, Hong CO (2020). Mechanism of cadmium immobilization in phosphate-amended arable soils. Appl Biol Chem..

[87] Loix C, Huybrechts M, Vangronsveld J, Gielen M, Keunen E, Cuypers A (2017). Reciprocal interactions between cadmium-induced cell wall responses and oxidative stress in plants. Front Plant Sci..

[88] Douchiche O, Rihouey C, Schaumann A, Driouich A, Morvan C (2007). Cadmiuminduced alterations of the structural features of pectins in flax hypocotyl. Planta..

[89] Parrotta L, Guerriero G, Sergeant K, Cai G, Hausman JF (2015). Target or barrier? The cell wall of early- and later-diverging plants vs cadmium toxicity: Differences in the response mechanisms. Front Plant Sci..

[90] Van De Mortel JE, Villanueva LA, Schat H, Kwekkeboom J, Coughlan S, Moerland PD (2006). Large expression differences in genes for iron and zinc homeostasis, stress response, and lignin biosynthesis distinguish roots of Arabidopsis thaliana and the related metal hyperaccumulator Thlaspi caerulescens. Plant Physiol..

[91] Van De Mortel JE, Schat H, Moerland PD, Van Themaat EVL, Van Der Ent S, Blankestijn H (2008). Expression differences for genes involved in lignin, glutathione and sulphate metabolism in response to cadmium in Arabidopsis thaliana and the related Zn/Cd-hyperaccumulator Thlaspi caerulescens. Plant Cell Environ..

