# Supplementary figures and images for: Global leaf and root transcriptome in response to cadmium reveals tolerance mechanisms in Arundo donax L
Source: BMC Genomics. 2022 Jun 8;23:427. doi: 10.1186/s12864-022-08605-6 (PMC9175368; doi:10.1186/s12864-022-08605-6)

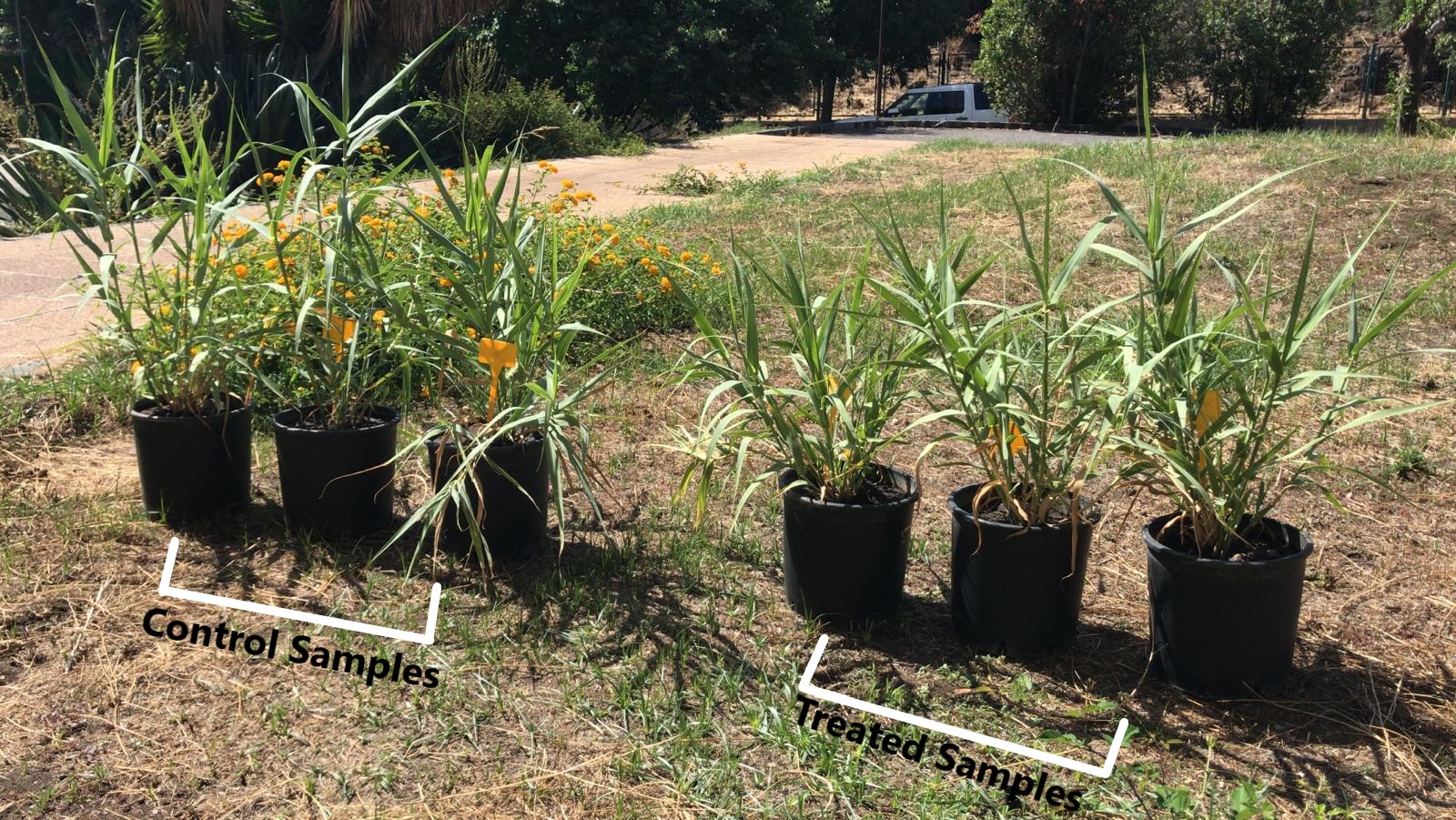


**Figure S1** - Picture of giant reed plants at sampling date (July 28th, 2020)

Supplement: Supplementary file 1 — Additional file 1: Figure S1. Picture of giant reed plants at sampling date (July 28th, 2020). [file 12864_2022_8605_MOESM1_ESM.docx]

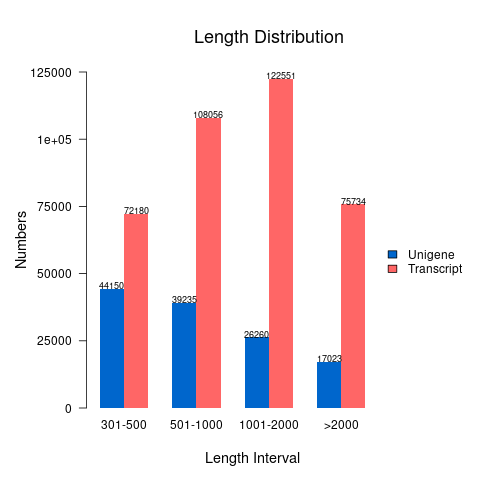


**Figure S4**. Length distribution of transcripts and unigenes

Supplement: Supplementary file 4 — Additional file 4: Figure S4. Length distribution of transcripts and unigenes. [file 12864_2022_8605_MOESM4_ESM.docx]

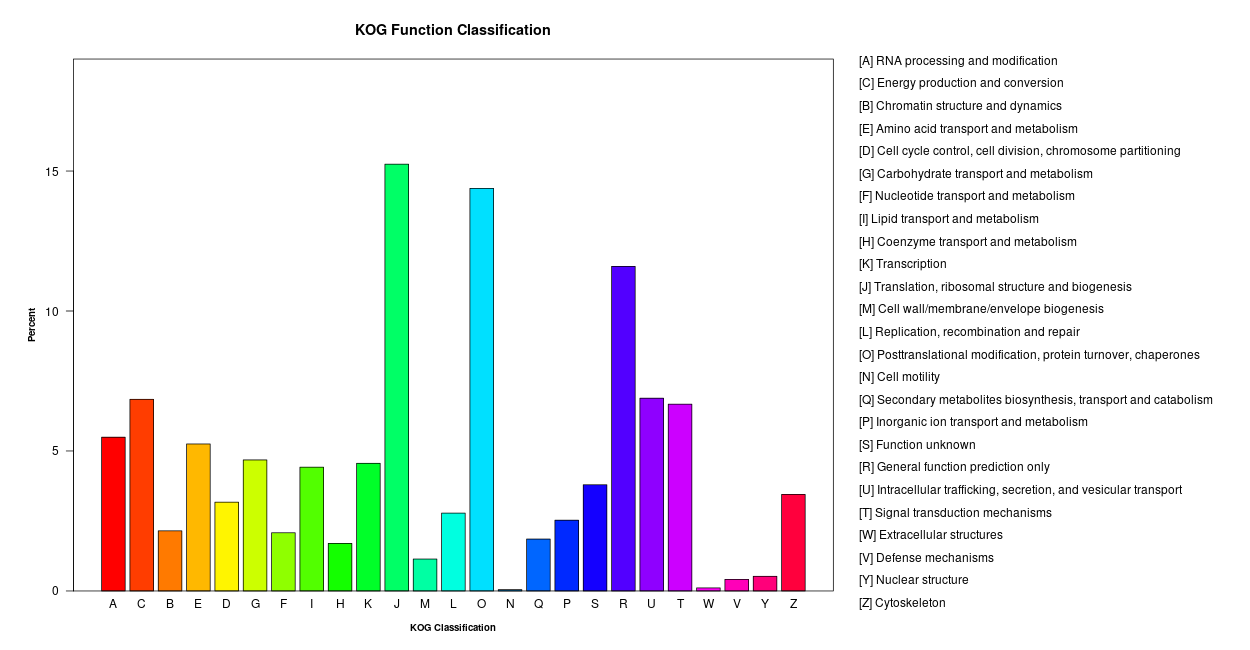


**Figure S8.** KOG functional classification

Supplement: Supplementary file 8 — Additional file 8: Figure S8. KOG functional classification. [file 12864_2022_8605_MOESM8_ESM.docx]
