# Supplementary material for: Global leaf and root transcriptome in response to cadmium reveals tolerance mechanisms in Arundo donax L
Source: BMC Genomics. 2022 Jun 8;23:427. doi: 10.1186/s12864-022-08605-6 (PMC9175368; doi:10.1186/s12864-022-08605-6)
Supplement: Supplementary file 2 — Additional file 2: Figure S2. Effect of cadmium treatment on morpho-biometric and physiological parameters of A. donax G10 ecotype. A) Main stem height per pot. B) Biomass dry weight. C) Net photosynthesis efficiency. [file 12864_2022_8605_MOESM2_ESM.pdf]

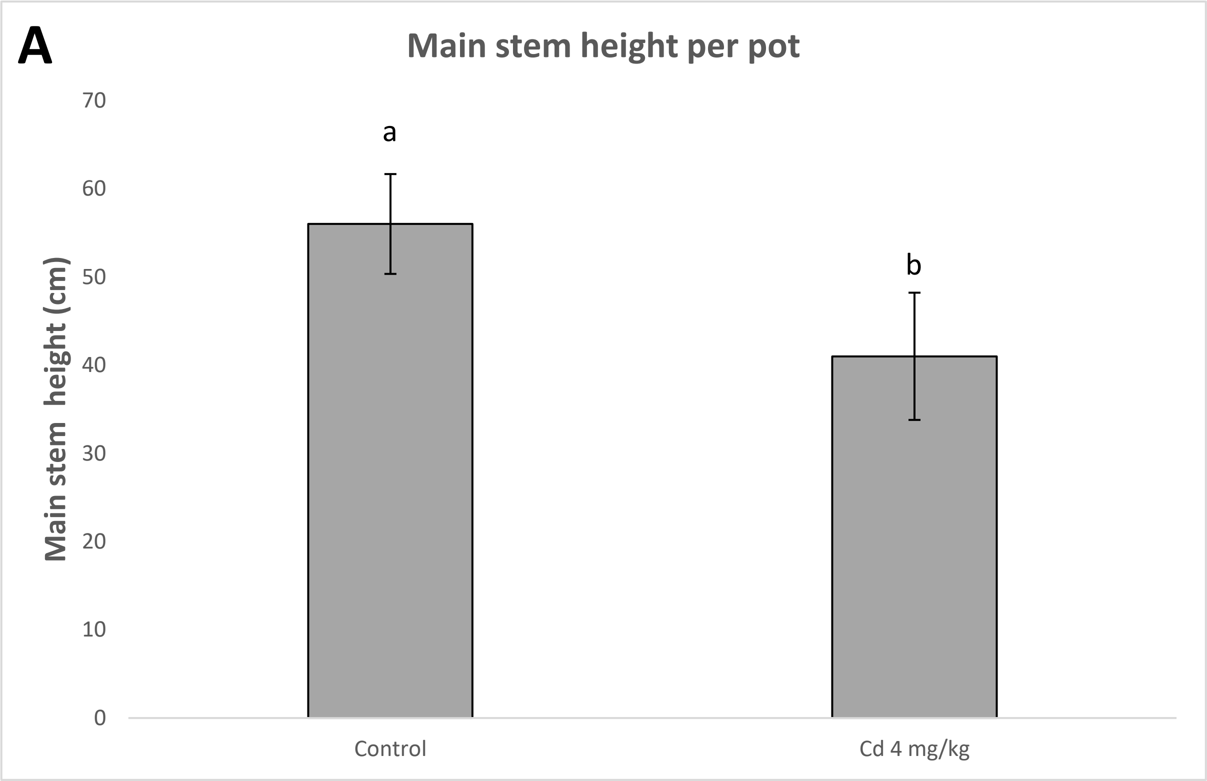
A
Main stem height per pot
Main stem height (cm)
70
60
50
40
30
20
10
0
a
b
Control
Cd 4 mg/kg

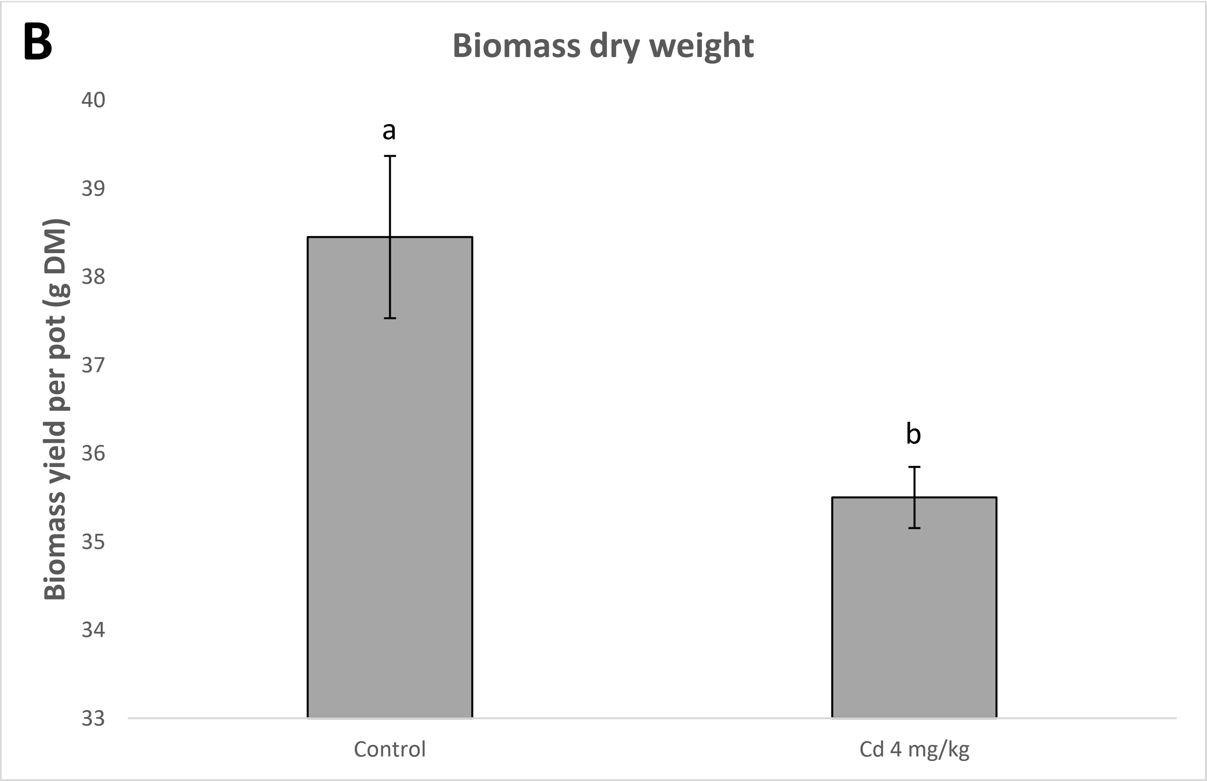
B
Biomass dry weight
Biomass yield per pot (g DM)
40
39
38
37
36
35
34
33
a
b
Control
Cd 4 mg/kg

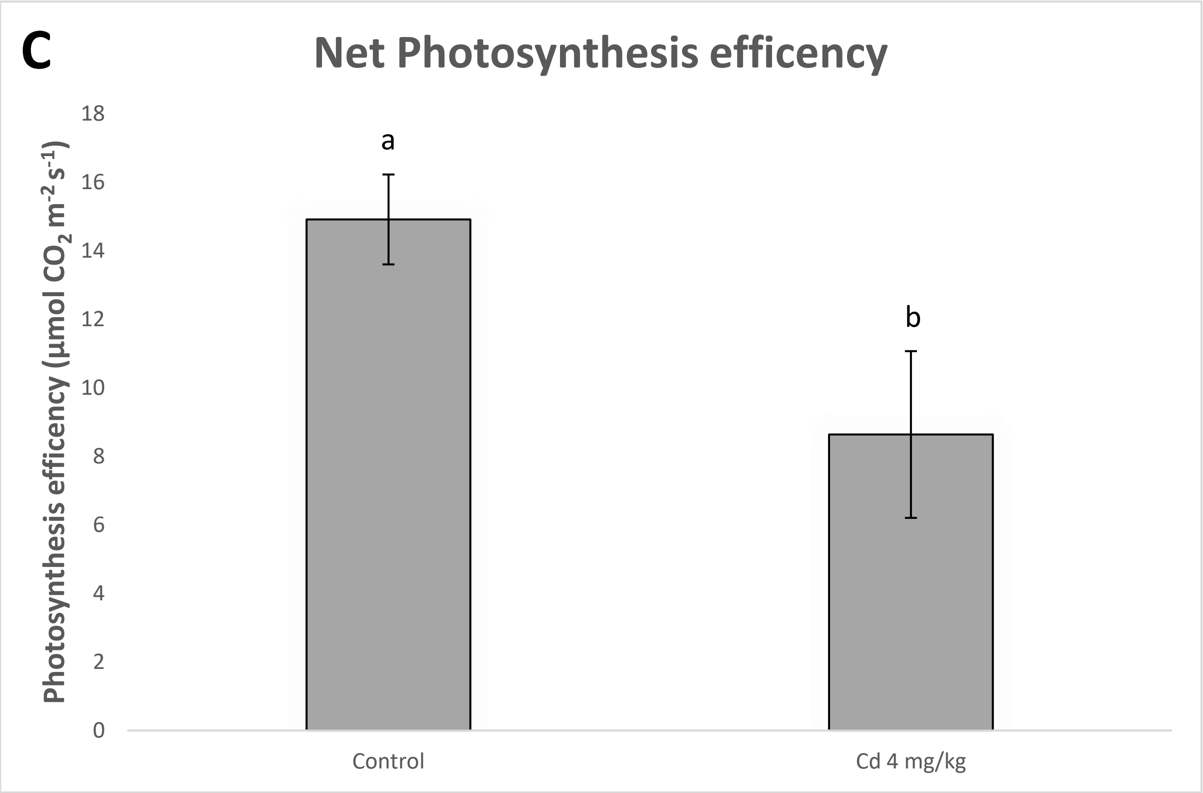

Figure S2. Effect of cadmium treatment on morpho-biometric and physiological parameters of G10 ecotype of *A. donax*. A) Main stem height per pot. B) Biomass dry weight. C) Net photosynthesis efficiency.
