# Supplementary material for: Global leaf and root transcriptome in response to cadmium reveals tolerance mechanisms in Arundo donax L
Source: BMC Genomics. 2022 Jun 8;23:427. doi: 10.1186/s12864-022-08605-6 (PMC9175368; doi:10.1186/s12864-022-08605-6)
Supplement: Supplementary file 3 — Additional file 3: Figure S3. Flowchart of sequencing and de novo assembly of A.donax leaf and root transcriptome under cadmium treatment. [file 12864_2022_8605_MOESM3_ESM.docx]

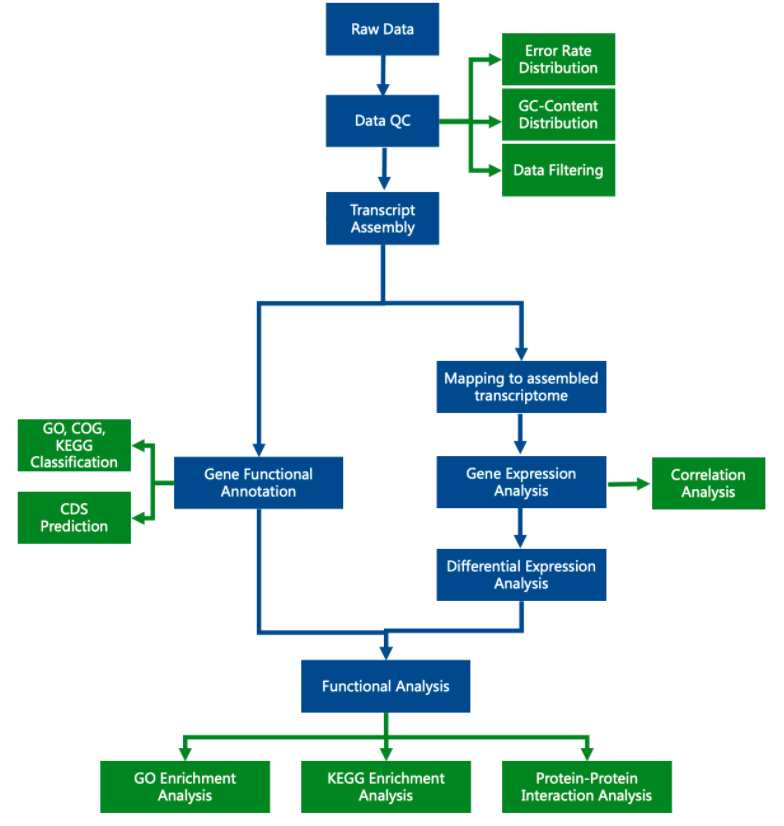


**Figure S3.** Flowchart of sequencing and *de novo* assembly of *A.donax* leaf and root transcriptome under cadmium treatment.
