# Supplementary material for: Global leaf and root transcriptome in response to cadmium reveals tolerance mechanisms in Arundo donax L
Source: BMC Genomics. 2022 Jun 8;23:427. doi: 10.1186/s12864-022-08605-6 (PMC9175368; doi:10.1186/s12864-022-08605-6)
Supplement: Supplementary file 6 — Additional file 6: Figure S5. Three-dimension PCA for RNAseq correlation. Principal Component coordinates were calculated using sample read counts. Figure S6 - Hierarchical clustering map for differential expression genes. [file 12864_2022_8605_MOESM6_ESM.docx]

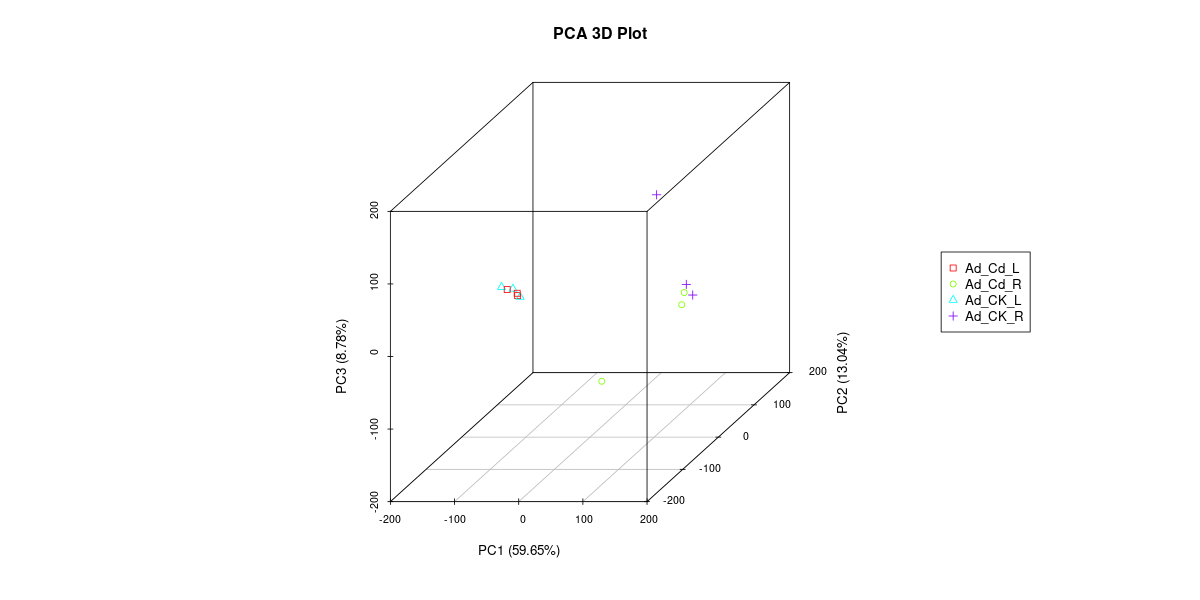


**Figure S5.** Three-dimension PCA for RNAseq correlation. Principal Component coordinates were calculated using sample’s readcount.


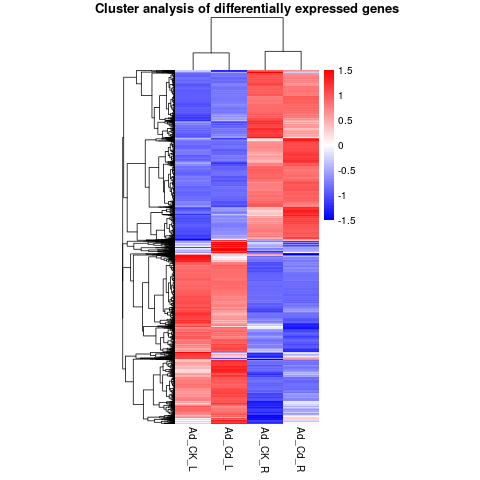


**Figure S6.** Hierarchical clustering map for differential gene expression
